# Joint representation and visualization of derailed cell states with Decipher

**DOI:** 10.1186/s13059-025-03682-8

**Published:** 2025-07-23

**Authors:** Achille Nazaret, Joy Linyue Fan, Vincent-Philippe Lavallée, Cassandra Burdziak, Andrew E. Cornish, Vaidotas Kiseliovas, Robert L. Bowman, Ignas Masilionis, Jaeyoung Chun, Shira E. Eisman, James Wang, Justin Hong, Lingting Shi, Ross L. Levine, Linas Mazutis, David Blei, Dana Pe’er, Elham Azizi

**Affiliations:** 1 Department of Computer Science, Columbia University, New York, NY 10027 USA; 2 Irving Institute for Cancer Dynamics, Columbia University, New York, NY 10027 USA; 3 Department of Biomedical Engineering, Columbia University, New York, NY 10027 USA; 4 Computational and Systems Biology Program, Sloan Kettering Institute, Memorial Sloan Kettering Cancer Center, New York, NY 10065 USA; 5 CHU Sainte-Justine Research Center, Montréal, QC Canada; 6 Department of Pediatrics, Université de Montréal, Montréal, QC Canada; 7 Immunology Program, Memorial Sloan Kettering Cancer Center, New York, NY 10065 USA; 8 Department of Medicine, Memorial Sloan Kettering Cancer Center, New York, NY 10065 USA; 9 Alan and Sandra Gerry Metastasis and Tumor Ecosystems Center, Memorial Sloan Kettering Cancer Center, New York, NY 10065 USA; 10 Human Oncology and Pathogenesis Program, Memorial Sloan Kettering Cancer Center, New York, NY 10065 USA; 11 Department of Cancer Biology, University of Pennsylvania, Philadelphia, PA 19104 USA; 12 Institute of Biotechnology, Life Sciences Centre, Vilnius University, Vilnius, 02158 Lithuania; 13 Department of Statistics, Columbia University, New York, NY 10027 USA; 14 Data Science Institute, Columbia University, New York, NY 10027 USA; 15 Howard Hughes Medical Institute, New York, NY 10065 USA; 16 Herbert Irving Comprehensive Cancer Center, Columbia University, New York, NY 10032 USA

**Keywords:** Deep generative model, Cell-state trajectories, Acute myeloid leukemia, Dimensionality reduction

## Abstract

Biological insights often depend on comparing conditions such as disease and health. Yet, we lack effective computational tools for integrating single-cell genomics data across conditions or characterizing transitions from normal to deviant cell states. Here, we present Decipher, a deep generative model that characterizes derailed cell-state trajectories. Decipher jointly models and visualizes gene expression and cell state from normal and perturbed single-cell RNA-seq data, revealing shared and disrupted dynamics. We demonstrate its superior performance across diverse contexts, including in pancreatitis with oncogene mutation, acute myeloid leukemia, and gastric cancer.

**Supplementary information:**

The online version contains supplementary material available at 10.1186/s13059-025-03682-8.

## Background

Single-cell genomic technologies have enabled the detailed characterization of cellular states in healthy and disease contexts, including cancer [1–5], inflammatory bowel disease [6, 7], and COVID-19 [8–10]. Within a tissue sample, cells exist in various cellular states and at various stages of differentiation. This variation allows a single snapshot of single-cell RNA sequencing (scRNA-seq) to reveal cellular states, their evolution, and characterize cell-state transitions by applying pseudotime inference approaches [11–15]. In particular, reconstructing how cells derail from normal to diseased states along a pseudotime axis promises to improve our knowledge of early disease stages, identify drivers of this derailment, and inform early detection and prevention strategies.

A prime example of derailed development occurs in acute myeloid leukemia (AML), a lethal cancer of the hematopoietic system. In AML, bone marrow hematopoietic stem and progenitor cells (HSPCs) acquire genetic and epigenetic abnormalities, leading to the accumulation of HSPC-like leukemic cells called “blasts” that fail to differentiate terminally. The origin of pre-leukemic and leukemic stem cell states in AML [5, 16] remains poorly characterized, which makes it difficult to target these cells and prevent disease recurrence [17–19]. Importantly, we do not know how specific mutations lead to distinct disease trajectories. These trajectories differ across patients [20] and can initiate from healthy states or pre-malignant and early malignant states such as clonal hematopoiesis and myelodysplastic syndromes [21–25]. Numerous other contexts, including disorders of embryonic development, neurodegenerative diseases, and T-cell exhaustion [26–29], require the accurate reconstruction of aberrant trajectories to understand their mechanisms.

However, existing methods often fail to reconstruct the order of events faithfully; linear approaches, such as principal component analysis (PCA), cannot capture biological complexity, while alternatives, such as neural networks, typically fail to represent the underlying biology and can mix the ordering of cell states. There is an urgent need for methods to accurately reconstruct the order of transcriptional events, precisely align trajectories, and compare disparate conditions, such as healthy to disease and control to genetic or chemical perturbation.

To compare trajectories, obtaining a faithful joint embedding and accurately visualizing the cellular relationships it represents is critical. This faithful embedding space can then be given as input to trajectory inference methods to extract and automatically compute trajectories. Embedding multiple samples, especially from heterogeneous cancers, is sensitive to minor differences in gene programs between samples, such that they typically fail to co-embed in a biologically meaningful way. Existing integration methods [30–37] are primarily designed for batch correction; they assume that samples share similar cell states and attempt to eliminate differences—including genuine biological differences, particularly for continuous and diverging trajectories—as technical effects. Moreover, most approaches compress information from thousands of genes into 10–50 factors that are independent, thereby neglecting dependencies between related biological processes (ignoring, for example, that divergent differentiation trajectories are related). The resulting latent spaces help annotate discrete cell states but often do not preserve gene-gene relationships and the order of cell states [38]. Furthermore, data is usually visualized by projecting latent embeddings onto two dimensions [39, 40], which can distort topology and obscure functional relationships [41]. These limitations highlight the need for approaches that address interpretability, preserve global geometry in the latent space, and enable visualization to better model trajectories perturbed by mutation, genetic manipulation, drugs, or disease.

In this work, we present Decipher (deep characterization of phenotypic derailment), an interpretable deep generative model for the simultaneous integration and visualization of single-cell data from disparate conditions. Decipher is a hierarchical model that learns two representations for each cell from the observed expression: a low-dimensional state (in an “intermediate” latent space of roughly ten dimensions, similar to existing methods [37, 42]), as well as a two-dimensional representation (“top” latent space) for visualization. Several design features allow this unifying model to characterize continuous trajectories more accurately: (1) it connects gene expression and the latent spaces with simple linear or one-layer neural network transformations to limit distortion, (2) the stacking of two latent spaces over gene expression space enables flexible capture of nonlinear mechanisms, despite the use of simple transformations, (3) it learns the dependency structure of cell-state latent factors with the top latent space embedding (unlike other methods, which assume that latent factors are independent), enabling the discovery of both shared and unique biological mechanisms from sparse trajectories, and (4) the 2D top latent space provides a direct visualization of the geometry learned by the model.

The hierarchical structure of Decipher allows for a comprehensive understanding of the relationships between gene expression, cell states, and their visual representation. We show that it is the only approach that preserves cell-state organization and continuity in synthetic data and demonstrate its substantial advantage for deriving insight from three disease contexts of increasing complexity—published data from a pancreatic ductal adenocarcinoma (PDAC) mouse model with an oncogenic mutation, new data from heterogeneous AML patient specimens, and a published patient cohort spanning two subtypes of gastric cancer.

## Results

### The Decipher method

Aligning trajectories from normal and perturbed contexts requires a joint representation that preserves the topology and order of cells along both trajectories without forcing artificial overlap. Decipher’s key assumption is that perturbed trajectories maintain shared transcriptional programs with normal trajectories for common processes, such as cell maturation. To create a joint representation that captures both biological differences and shared mechanisms, Decipher employs a hierarchical model featuring two levels of latent representations, each with its own encoder and decoder networks. This unique architecture allows for correlation between some latent factors, enabling the identification of shared gene programs that would be missed under the standard requirement for independence. Decipher uses simple linear transformations and single-layer neural networks to connect all representations within a unified probabilistic framework, making it sufficiently flexible to learn nonlinear mechanisms while imposing a rigid inductive bias that prevents arbitrary distortion of the global geometry.

**Fig. 1:**
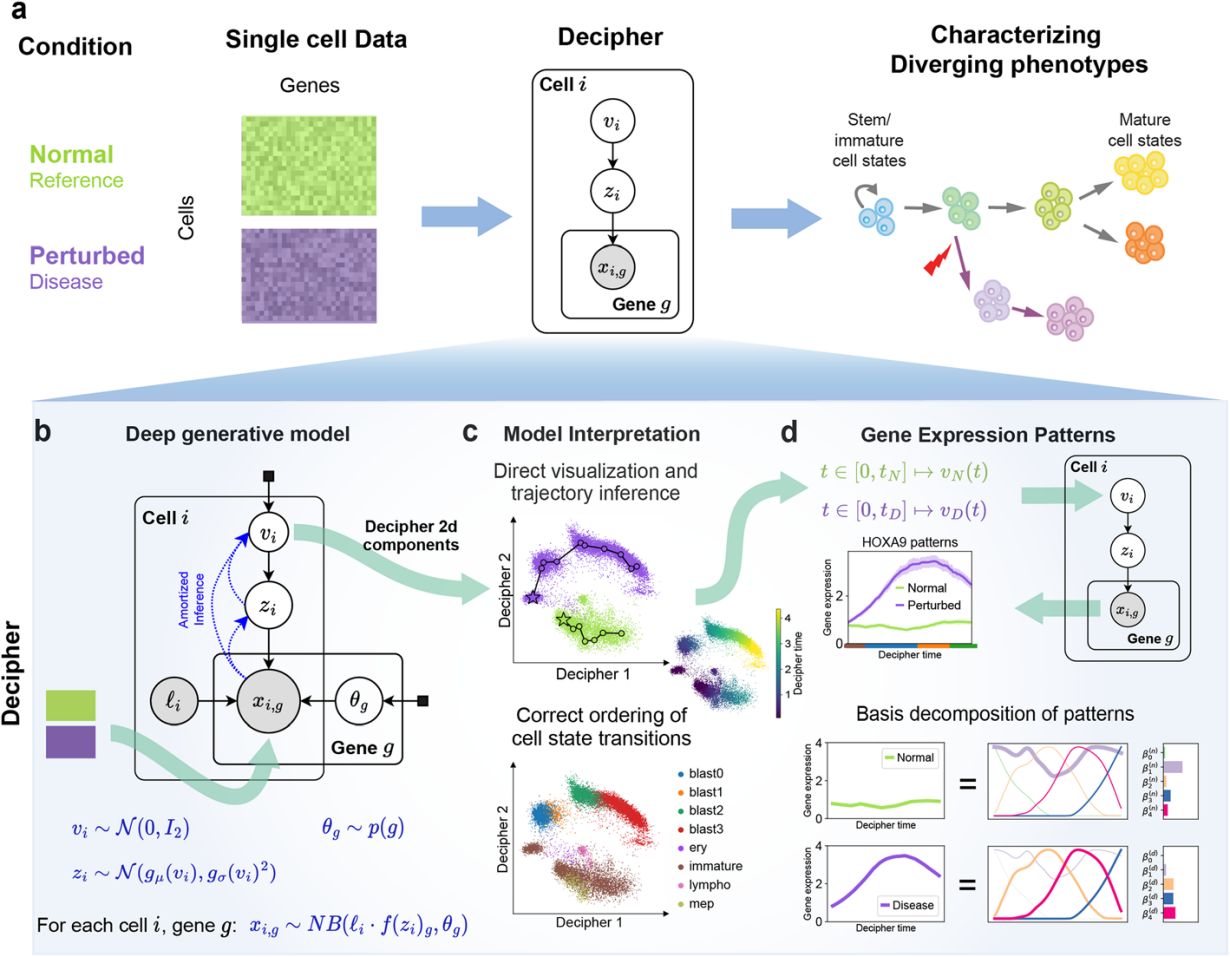
Overview of the Decipher framework. **a** Decipher accepts multiple single-cell datasets (e.g, a normal reference and a perturbed condition), which its probabilistic model represents in a hierarchical shared latent space without removing biological differences. The latent space reveals shared cell-state transitions and characterizes diverging phenotypes. **b** Decipher’s generative model has three levels of cell representations (distributions shown at bottom): 2D Decipher components v, latent factors z, and gene expression x. Decipher components summarize heterogeneous cell states and are used to directly visualize the latent space. **c** Example of Decipher space visualization, colored by the dataset of origin (normal or disease) and by independently annotated cell states. Two distinct trajectories (lines and circles; stars depict start) are computed in the Decipher space. **d** Gene expression patterns are computed along each trajectory using Decipher’s decoders mapping v to x and then decomposing into representative patterns (basis). The corresponding weights are used to compare patterns between the two contexts to pinpoint disrupted and conserved genes

To enable correlated latent factors, Decipher extends the successful single-cell variational auto-encoder (VAE) architecture [37, 42–45] into a deeper generative model inspired by the deep exponential family [46, 47]. In Decipher, each cell has not one but two complementary latent representations (Fig. 1a, b). First, we embed cells in a two-dimensional representation that encodes global cell-state dynamics. We refer to this high-level embedding as the Decipher space and its two dimensions as Decipher components. Decipher components represent the dominant axes of variation in the data, typically progression (maturation) and derailment (degree of deviation from a normal process, e.g., in disease). Then, we generate a higher-dimensional representation conditional on the two Decipher components, designed to capture more refined cell-state information. We call the space of refined representations the latent space and refer to its dimensions as latent factors. The latent space is similar in principle to previous VAEs [37, 43, 44], except that the design of Decipher, which conditions the latent space on the Decipher components, enables dependencies between latent factors. Finally, Decipher generates the denoised gene expression of each cell from its inferred cell state (Fig. 1b and Methods section).

This three-step generative process interpolates between different degrees of non-linearity and generates latent factors whose dependencies are shaped by high-level Decipher components, offering major advantages for interpretation. Cell representations can be visualized in 2D directly from Decipher components, eliminating the need for further dimensionality reduction with methods such as t-distributed stochastic neighbor embedding (tSNE) or uniform manifold approximation and projection (UMAP), whose usage is a subject of debate [41] (Fig. 1c). Within the Decipher space, derailed trajectories can be constructed along a joint pseudotime that we call Decipher time.

In addition, Decipher is formulated to give explicit mapping functions between gene space and Decipher space, enabling a straightforward reconstruction of gene expression anywhere in Decipher space (Methods section) and enabling the imputation of gene trends along the entire trajectory (Fig. 1d). This is particularly useful for determining gene expression levels in sparse locations of the Decipher space with few observed cells. It also enables the reciprocal computation of Decipher components (and straightforward visualization) for any cell with measured gene expression. In contrast, there is no explicit mapping between UMAP space and the gene expression space. Decipher offers a unique framework for dimensionality reduction, 2D visualization, trajectory alignment, and characterization of cell state transitions.

### Decipher preserves sparse simulated trajectories

**Fig. 2:**
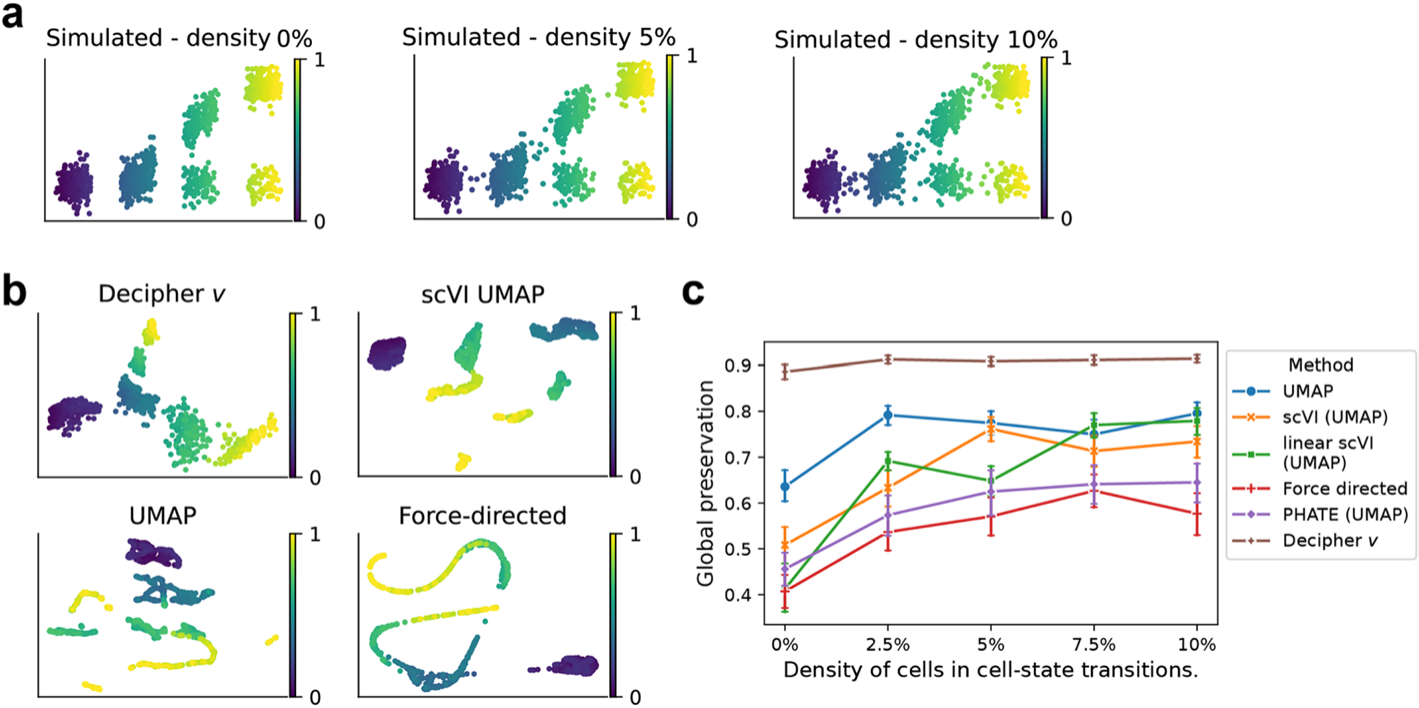
Comparison of methods on simulated data. **a** Simulated cell states along diverging trajectories with downsampled (0% to 10% cell density) cell-state transitions. Color gradient represents ground-truth simulated pseudotime. **b** Latent spaces learned by different methods on ground truth data with a cell-state transition density of 0%. Only Decipher preserves the global order of cell states. **c** Global preservation of five independent random datasets across a range of cell-state transition densities, and by method (Methods section; 1 indicates best preservation). Error bars, s.d. Decipher best preserves the global order of cell states for all cell-state transition densities

To benchmark Decipher against alternative methods, we simulated ground truth continuous cell-state trajectories by randomly sampling two-dimensional vectors (representing cell states) along a forked trajectory containing regions of low (0% to 10%) sampling density (Fig. 2a). The sparsely sampled regions reflect realistic variation in data collected from snapshots of a stepwise differentiation process [14, 48]. We transformed ground truth cell-states into gene expression using random neural networks, similar to scVI’s generative process (Methods section), then visualized the data using popular dimensionality approaches (force-directed layout [40], UMAP [49], PHATE [50], scVI [37]), and measured how well they recover the true organization of cell states.

Only Decipher produced visualizations that reflect the two trajectories in the correct order (Fig. 2b). Errors made by the other tested methods, such as the proximity of initiating and terminal cells in the scVI latent space visualized with UMAP, are caused by low cell densities in transitional regions. Although cell-state transitions are common and important in biology, current methods are only designed to preserve distances in locally continuous data and thus lose the global geometry of cell states. We quantitatively evaluated the latent representation using a global preservation metric [41], which measures the accuracy of cell-state ordering by first computing a nearest-neighbor graph on ground-truth data clustered into cell states, then determining whether neighbors are retained in the learned visualization (Methods section). Decipher space exhibits much greater global preservation than the other methods across a realistic range [51] of transitional cell densities, with the most pronounced improvement in lowly sampled regimes (Fig. 2c).

### Decipher improves the interpretation of oncogenic trajectories

**Fig. 3:**
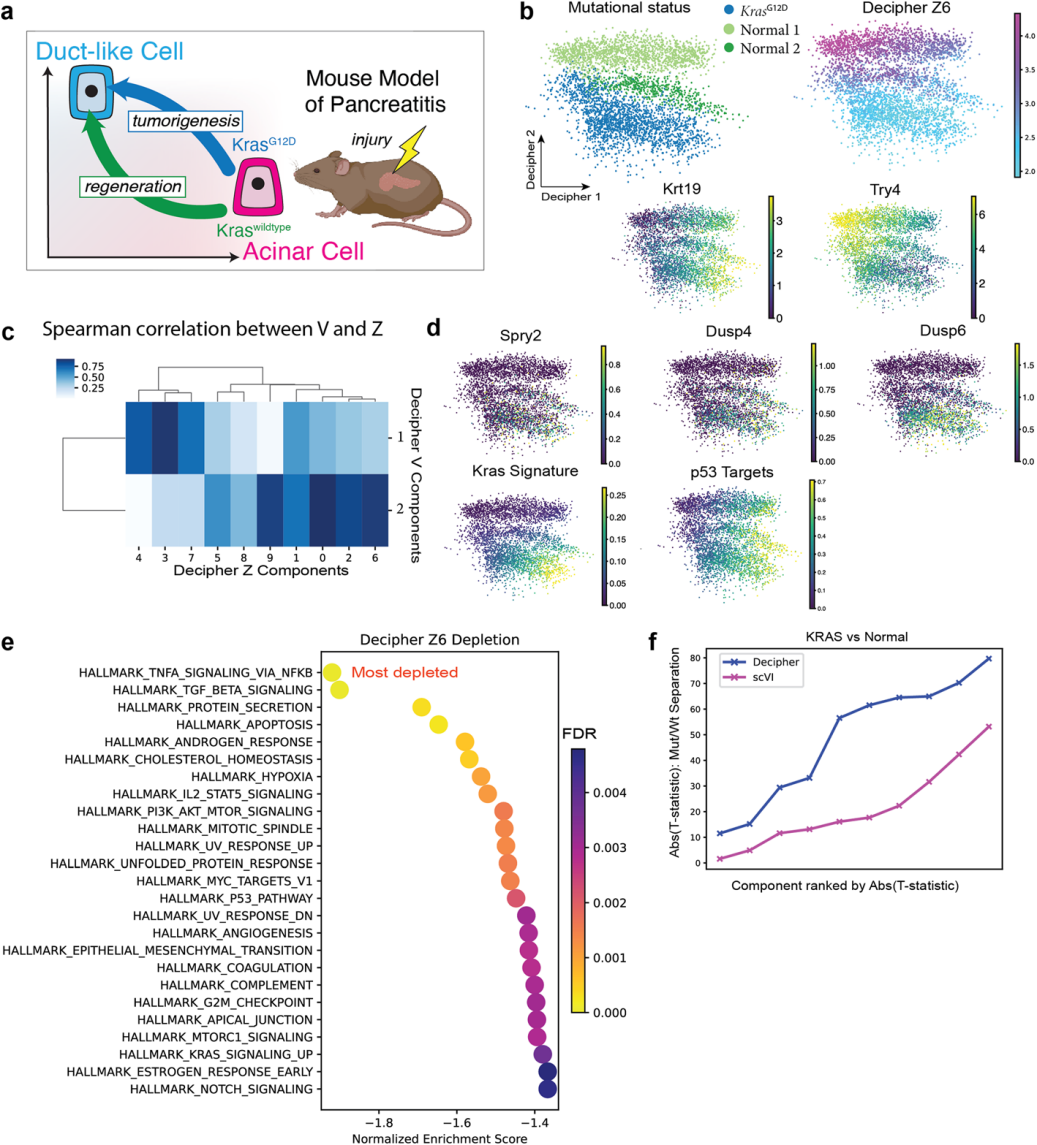
Decipher delineates the impact of *Kras* mutation on pancreatic regeneration. **a** Model of pancreatitis in mice. Injury drives acinar cells to ductal-like cell states, aiding regeneration in wild type but promoting tumorigenesis in an oncogenic *Kras* background. **b** Decipher 2D space colored by *Kras* mutation status, latent factor z6 loading, or acinar (*Try4*) or ductal (*Krt19*) marker expression. **c** Pearson correlation between Decipher components (v) and latent factors (z). **d** Decipher 2D space colored by expression of the *Kras* mutational signature, p53 targets, and *Kras* targets (*Dusp4*, *Dusp6*, *Spry2*). **e** Pathways depleted in Decipher z6 (top 25 selected from the 42 pathways with FDR < 0.25). **f** Absolute value of the t-statistic comparing the distributions of each latent factor in *Kras*-mutated (*Kras*$^{G12D}$) versus normal cells for Decipher and scVI, sorted from least to greatest t-statistic

We show how Decipher can characterize the impact of oncogenic *Kras* mutation on pancreas regeneration in mice [52, 53]. Following injury, wild-type epithelial cells undergo physiological metaplasia and regeneration, whereas *Kras*-mutated cells enter a premalignant state that begins expressing oncogenic programs, presaging cancer [54] (Fig. 3a). We apply Decipher to the data collected by [52, 53]. Decipher’s 2D space successfully separates wild-type (“normal”) from mutant conditions and organizes cells into three smooth visual trajectories corresponding to two normal conditions and a *Kras*-mutated condition (Fig. 3b).

Importantly, Decipher 1 captures the well-known process of acinar-to-ductal metaplasia (ADM), which appears as a smooth progression from acinar (*Try4*+) to ductal (*Krt19*+) cells (Figs. 3b and S1a; Methods section). ADM is a normal regenerative response to injury in both healthy and disease systems [55, 56], highlighting Decipher’s ability to model shared cellular processes. At the same time, Decipher 2 delineates the derailed trajectory to *Kras*-induced premalignant states, separating trajectories by the degree of deviation from normal while maintaining their alignment to the shared ADM process. Decipher correctly identifies that one normal condition is more similar to the *Kras*-mutated condition, as supported by the shared expression of important regulators such as the *AP1* factors, likely induced by stress, which also occurs during normal regeneration (Additional file 1: Fig. S1b). By faithfully representing the global geometry of the data, Decipher thus generates highly interpretable 2D components (*v*).

The latent factors (*z*) learned by Decipher offer further insight into the derailment by distinguishing key cell populations (Additional file 1: Fig. S1c) and revealing which genes inform these distinctions. We identified the top factor separating *Kras*-mutant and normal cells. We found that this top factor (z6) highlights the *Kras*-mutated population and is strongly driven by Decipher 2 (Fig. 3b, c and Methods section). To identify genes associated with z6, we computed the correlation between the expression of genes across all cells and latent factor weights. Notably, *Kras* target genes *Dusp6*, *Dusp4*, *Spry2*, and *Spry4* were ranked significantly higher, by correlation with negative z6, than the ranking distribution of all genes (*p* = 0.006, Wilcoxon rank sum test; Fig. 3d and Additional file 3: Table 1). Gene set enrichment analysis on factor z6 using these correlation-based gene rankings identified 42 significantly enriched pathways (FDR *q* < 0.25; Additional file 3: Table 2), including TNF, TGFB, MYC, and p53 pathways associated with tumorigenesis (Fig. 3e; Additional file 2: Table S2). Finally, a *Kras* mutational signature derived from bulk data [52] increases along the Decipher 1 axis and is only enriched in the *Kras* mutated population of cells, while p53 targets [57] increase along all three trajectories (Fig. 3d), reflecting p53’s intact status at this premalignant stage. These results support Decipher’s ability to dissect premalignant states and clearly illustrate the derailment from normal regeneration caused by a single oncogenic mutation. Notably, while gene set enrichment analysis on the latent factor of scVI highlighting the *Kras*-mutant population returns similar hits (Additional file 1: Fig. S1e), the separation between *Kras* mutated and wild-type cells in the z factors of Decipher is generally greater than the separation in the latent space of scVI (Fig. 3f). This result suggests that Decipher may be more successful in capturing the distinction between key biologically distinct populations in its latent spaces.

Decipher can recover more pathways associated with the *Kras*-deviated trajectory since it allows dependencies between factors, which helps identify shared and distinct features of each sample (Fig. 3c). By simultaneously capturing the shared physiological metaplasia (via Decipher 1) and the distinct oncogenic derailment (via Decipher 2 and z6), Decipher provides a robust framework for interpreting both normal and disease trajectories. This dual capability for modeling and visualizing shared and distinct processes helps elucidate how mutations like *Kras* alter normal cell-state transitions and initiate oncogenic programs.

### Leukemic derailment in AML initiates from immature cells

Next, we applied Decipher to investigate the complex and poorly understood derailment of early leukemic cells in AML. We collected 104,116 single-cell transcriptomes from bone marrow specimens of a cohort of AML patients bearing *TET2* epigenetic mutations (*n* = 12), with and without *NPM1* mutations, as well as a healthy donor as reference (Additional file 3: Table 3). *NPM1* is among the most commonly mutated genes in AML (20–30% of cases), yet its role in leukemogenesis [58] is unknown. *NPM1*^mut^ AML often co-occurs with mutations in the epigenetic modifiers *TET2* and *DNMT3A*. These genes are known drivers of clonal hematopoiesis, a condition associated with an elevated relative risk of progression to myeloid malignancy in older adults [59]. The epigenetic mutations likely originate in pre-leukemic hematopoietic stem cells (HSCs) [60], facilitating the development of AML after *NPM1* mutation [61]. However, the transcriptomic consequences of *NPM1* mutations and the influence of pre-existing epigenetic abnormalities remain unclear.

**Fig. 4:**
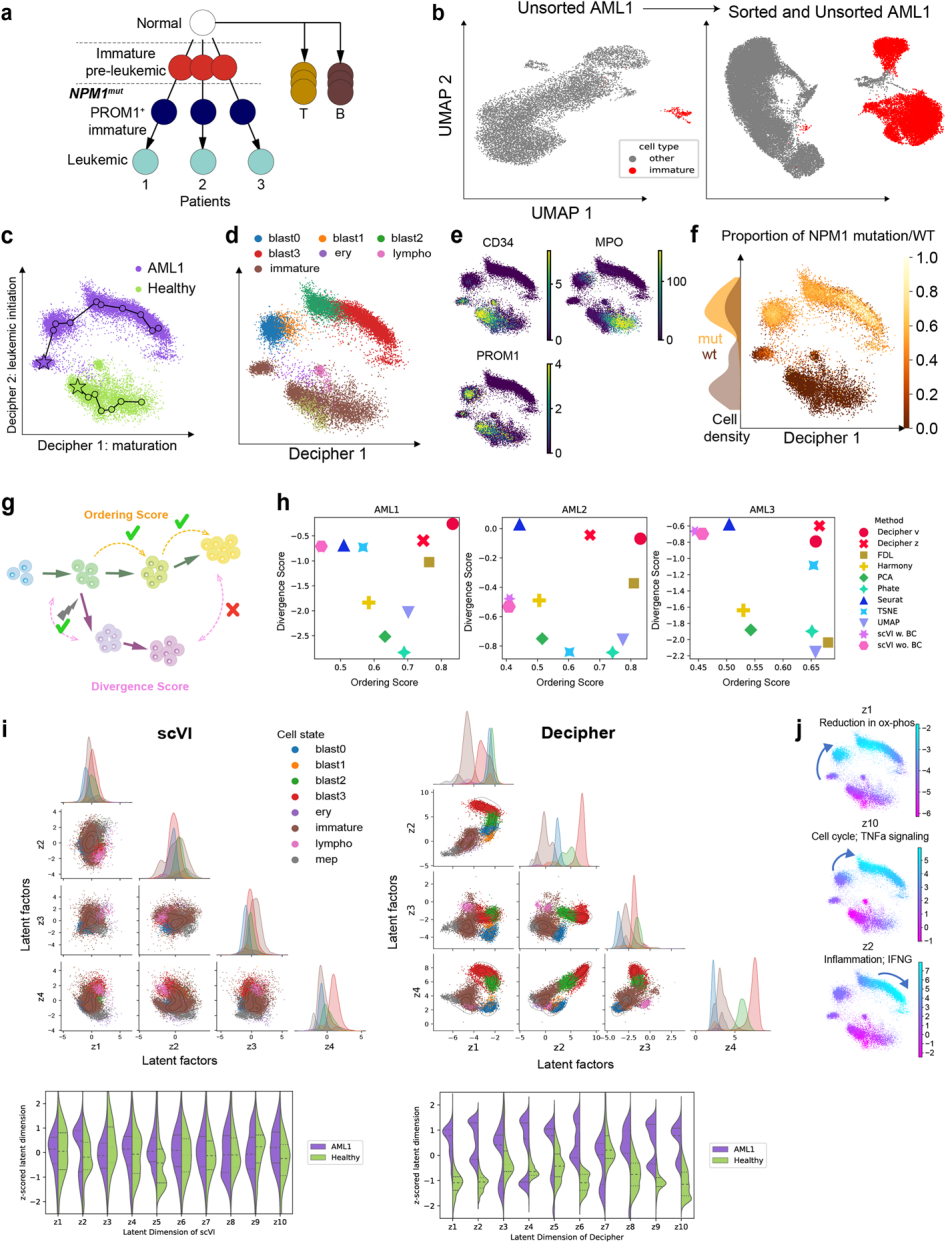
Decipher reconstructs derailed myeloid developmental trajectories in AML. **a** AML is characterized by patient-specific trajectories with similar immature wild-type cells, shared initial states, and divergent trajectories to terminal states following *NPM1* mutation. **b** UMAP projection of AML1 single-cell transcriptomes with (right) and without (left) the inclusion of sorted *CD34*^*+*^/*PROM1*^*+*^ cells to enrich for HSC-like cells. **c**–**f** Decipher space embedding of 37,395 sorted cells from patient AML1 and a healthy donor, colored by the sample of origin (**c**), cell states (**d**), key cell markers (**e**), and *NPM1*^mut^-to-*NPM1*^wt^ proportion (**f**) (Methods section). Decipher 1 organizes cells along maturation, and Decipher 2 along leukemia initiation axes. Lines and circles represent post-analysis trajectories; stars indicate initiating states. **g**, **h** Two metrics that measure a latent space’s interpretability and faithfulness to underlying biology (**g**; Methods section) used to benchmark Decipher against dimensionality reduction and harmonization methods (**h**). **i** Comparison of scVI [37] (left) and Decipher (right) latent spaces for pairs of the first four latent dimensions colored by cell state (top; the other latent dimension pairs give similar results) and for all latent dimensions colored by sample origin (bottom). The scVI latent space collapses biological differences while Decipher preserves them. **j** Decipher space colored by latent factors z1, z10, and z2, each capturing a different state transition

Consistent with prior studies [20, 62], we found significant inter-patient heterogeneity in leukemic blast cells (Additional file 1: Fig. S2a, b), which is unlikely due to technical effects given that lymphocytes are well mixed. Surprisingly, the most immature, HSC-like cluster (448 cells; 0.4% of total) is the top non-lymphoid cluster shared by most patients (Additional file 1: Fig. S2a, c). The phenotypic similarity of immature leukemic or pre-leukemic cells across patients contrasts with the heterogeneity of leukemic cells, motivating the reconstruction of patient-specific trajectories diverging from normal HSPCs (Fig. 4a). However, our samples contained too few HSC-like cells to characterize *NPM1*-mediated derailment effectively. To address this, we identified two differentially expressed surface markers from our cohort data, *CD34* (log fold change: 6.67, adjusted $p <$1e−6) and a novel maker, *PROM1* (*CD133 *[63]; log fold change = 7.23, adjusted $p<$ 1e−6), and used them to enrich the immature population in *NPM1*^mut^ patients. Sorting for cells expressing either marker expanded the target population from 179 to 13,210 cells in patient AML1 (Fig. 4b), and a total of 29,266 immature cells from AML1–AML3 (Methods section). *NPM1* mutations can be detected directly from scRNA-seq data [64] because the vast majority occur at the 3′ end of the gene [58] in AML (Additional file 2: Table S1). The expanded HSC-like population revealed cryptic heterogeneity in both *NPM1* mutation status and maturation (Additional file 1: Fig. S2d, e). Our data spanning leukemic progression, especially the rare early stages around *NPM1*^mut^-mediated derailment, thus poised us to ask exactly when and how cells diverge from myeloid differentiation in normal HSPCs.

### Decipher reconstructs maturation and derailment in AML

We applied Decipher to integrate data from a healthy individual with data from each patient AML1, AML2, or AML3 separately (Figs. 4c, S3a, b). For each patient, we found that Decipher 1 and 2 faithfully represent the shared processes of cell maturation and disease derailment, respectively. Specifically, Decipher 1 captures the stepwise maturation of leukemic cell states from immature to blast 0 through 3 in a leukemic derailment trajectory (Figs. 4d, S3a, b). It also captures myeloid differentiation, as determined by loss of *CD34* (stem marker) and gain of *MPO* in a normal cell-state progression trajectory (Fig. 4e). Decipher 2, in contrast, represents an axis of leukemic initiation and progression that can be further interpreted using *NPM1* mutation status. We found a subset of pre-leukemic immature *NPM1*^wt^ cells close to healthy HSCs, and an *NPM1*^mut^ progenitor-like population (blast0,1) that lies between *NPM1*^wt^ and leukemic cell states resembling myeloid-committed cells (blast2,3) in all three patients (Figs. 4d, f and S3a, b). The increase in *NPM1*^mut^ cell fraction and upregulation of PROM1 along Decipher 2 confirm that it distinguishes leukemic from normal cell states (Fig. 4e, f). Thus, similar to the pancreatitis example, the Decipher 2D space represents major axes of biological variation and preserves global relationships between cell states in the complex context of AML. It correctly places *NPM1*^wt^ leukemic cells closest to normal (Fig. 4f) and orders leukemic blasts by maturation (Additional file 1: Supplementary Information). We further find that the relative ordering of cell states is robust to the choice of gene-filtering before training Decipher (see Methods section), and is correct even when running Decipher with all the genes, without any gene filtering (Additional file 1: Fig. S12). Decipher also identifies trajectories with both shared and distinct features for patients AML1 to AML3. AML2 has a larger gap between *NPM1*^wt^ and *NPM1*^mut^ cells in Decipher space (reinforced by the absence of detected blast 0 cells in this patient). Branching in AML3 occurs during blast1 rather than before blast0, suggesting later derailment in this patient than in AML1 or AML2 (Additional file 1: Fig. S3a, b). To reinforce these patient-specific differences, we use Decipher to integrate cells from AML1, AML2, and AML3 (Additional file 1: Fig. S3c) and determine if these trends are shared. This analysis reveals a correct alignment of cell states between patients, including preserving the gap in AML2 corresponding to the missing blast 0 population.

To further characterize early disease processes, we used gene set enrichment analysis (GSEA) on cells projected onto the Decipher 2 derailment axis. We identified TNFa signaling, inflammatory response, IL6/JAK/STAT3 signaling, IFNg response, and KRAS signaling pathways (Additional file 3: Table 4 and Methods section). These findings agree with the well-elucidated role of *Tet2* in repressing IL6 transcription [65] and with the association of *Tet2* loss-of-function with the accumulation of inflammatory myeloid cells in conditions from clonal hematopoiesis-related atherosclerosis [66] to AML [67].

In contrast to Decipher, we found that the visualization approaches tSNE [68], UMAP [49], and force-directed layout (FDL) [40] fail to capture the global data geometry, the expected overlap in immature cell states, or the order of blast maturation stages. Furthermore, data integration methods tend to force cell states to overlap—including leukemia cells and normal HSPCs—and thus cannot be used to characterize derailed differentiation (Additional file 1: Fig. S4). To systematically benchmark the ability to characterize derailed trajectories, we defined two metrics that evaluate biological faithfulness to AML derailment (Fig. 4g and Methods section). The ordering score evaluates whether each method’s latent space correctly orders cell states by the maturation stage. The divergence score assesses the preservation of biological differences by rewarding immature cell proximity and penalizing the mixing of normal and disease terminal states. We applied these metrics to a range of visualizations (FDL [40], UMAP [49], tSNE [68], PHATE [50]), dimensionality reduction (PCA), and integration methods (Seurat [31], scVI [37], Harmony [69]). Decipher components and latent factors scored highest in both metrics for all three patients, demonstrating that it balances between integrating across conditions and preserving their unique geometries and cell states (Fig. 4h).

### Decipher can represent correlated biological mechanisms

The hierarchical dimensionality reduction in Decipher confers more expressive factors that cannot be attained by simply increasing the number of latent factors in other methods [46]. Since Decipher’s latent factors can be correlated (Fig. 4i), they are able to capture overlapping transcriptional programs between trajectories and shared mechanisms underlying consecutive cell state transitions. For example, factor z7 is mainly encoded with Decipher 1 (Additional file 1: Fig. S5a) and represents common trends along both normal and AML trajectories (Additional file 1: Fig. S5b).

Latent factors can also highlight features that distinguish normal and perturbed states. For example, factors z1 and z2 are positively correlated in immature cells but negatively correlated in blasts (Figs. 4i, S5c). They represent different transitions along AML derailment—z1 is highest early in blast formation (blast0, 1, and 2), z10 marks an intermediate (from blast1 to 2), and z2 marks the final stage of leukemic maturation (blast3) (Fig. 4j). GSEA further supports these interpretations. Factor z1 is enriched for reduction in oxidative phosphorylation, a pathway that is altered in leukemic stem cells [70], while z10 and z2 are enriched for TNF$\alpha$, IFNg and inflammation [71, 72] (Fig. 4j and Additional file 3: Table 4). Indeed, the enrichment of IFNg and inflammation in z2 (Additional file 3: Table 4) is expected as it captures most mature myeloid/monoblastic cells [71, 72]. Decipher thus enables a more comprehensive and nuanced understanding of cellular dynamics, especially when integrating normal and perturbed conditions.

Decipher’s unique ability to model correlated latent factors avoids the requirement for independent factors, which can remove biological differences between cell states. For example, scVI collapses the healthy and AML conditions onto each other in every latent dimension (Fig. 4i), deforming the geometry and disrupting continuous trajectories (Additional file 1: Fig. S4b).

### Gene patterns along Decipher trajectories reveal altered regulation in AML

To uncover gene expression dynamics along cell maturation and disease derailment trajectories visualized by Decipher (Fig. 4c, d), its decoders can directly transform any cell state in Decipher space, including sparsely sampled states, to their corresponding gene expression mean and variance.

**Fig. 5:**
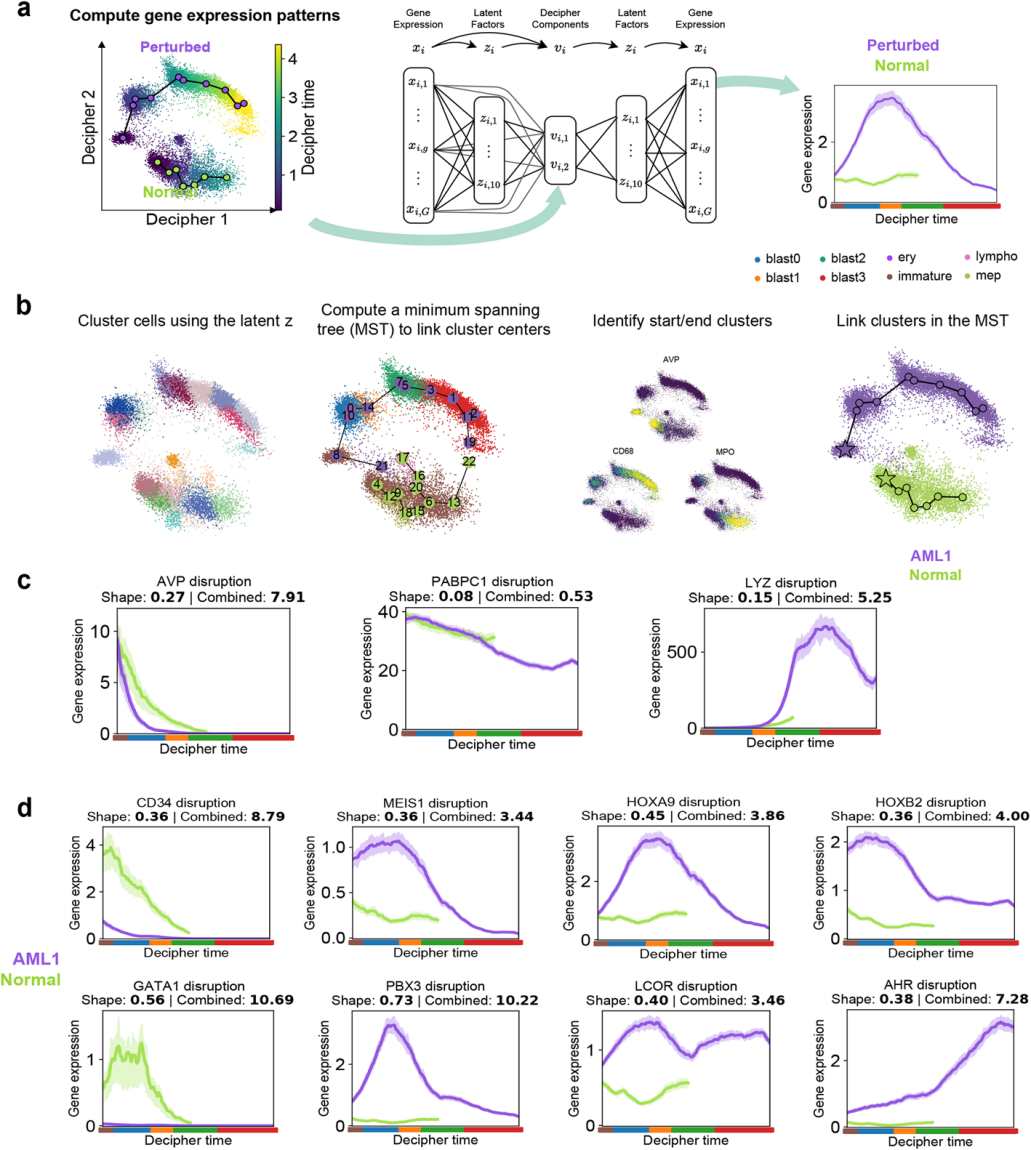
Reconstructing gene expression patterns and characterizing the regulatory landscape in AML compared to healthy HSPCs. **a** The Decipher generative model reconstructs gene expression along each trajectory directly from the 2D Decipher representation. **b** Steps involved in trajectory inference, from left to right, based on the inferred representation from Decipher (Methods section). **c** Reconstructed expression of stemness and differentiation markers for each trajectory along Decipher time. Shaded bands represent the interquartile range of Decipher model uncertainty (Methods section). **d** Reconstructed gene expression dynamics of *HOXA9* and *MEIS1* (known deregulated TFs) and other disrupted TFs. Solid lines show inferred mean, shaded areas reflect $\pm 1$ s.d.

We constructed paths along the visual trajectories in Decipher space and computed expression along these paths to obtain gene patterns (Fig. 5a and Methods section). Explicit trajectories can be defined manually or obtained with any trajectory inference method. The resulting coordinates along those paths define a pseudotime called *Decipher time* (Fig. 5a). Importantly, since different conditions are integrated into the same joint space, the trajectories have comparable Decipher time, with no further alignment needed to compare trajectories. The gene patterns inferred by the Decipher decoder can thus be directly compared “out of the box” without the additional challenging trajectory alignment required by standard integration methods. The similar patterns of key developmental markers *CD34 *[65], *AVP* (stemness), *PABPC1* (protein synthesis in HSC differentiation), and *LYZ* (myeloid differentiation) in aligned segments of the trajectories defined in the integration of AML and healthy (Fig. 5b), as well as the patterns of key genes in the combination of AML patients across the cohort (Additional file 1: Fig. S3c), confirm that the inferred pseudotime is comparable between datasets.

We implemented a simple method that clusters cells in the latent space, generates a minimum spanning tree to link clusters, and then interpolates between clusters in Decipher space (Figs. 5b, S6 and Methods section). As an alternative, one can use any existing trajectory inference method with the Decipher space as input instead of their default dimensionality reduction. We show examples with Monocle [66], Slingshot [12], and PAGA [67]. Monocle and Slingshot recover equivalent trajectories when they are run on the Decipher space (Additional file 1: Fig. S6e, i). In contrast, they fail to recover biologically meaningful trajectories when they are used with their default embedding strategy like PCA or UMAP (Additional file 1: Fig. S6c, g). For example, Monocle without Decipher incorrectly connects the blast 3 cells to immature cells within the AML patient and fails to link blast 1 to blast 2 cells (Additional file 1: Fig. S6c). Slingshot without Decipher produces a degenerate trajectory, implying that blast 3 cells mature into blast 1 cells (Additional file 1: Fig. S6g). The proximity of the blast 3 cells to the immature cells leads Slingshot to mix those two cell states, thereby misaligning healthy and AML trajectories. As a result, the gene patterns we would obtain with those incorrect trajectories are also biologically inaccurate and misleading (Additional file 1: Fig. S6d, h). For instance, computing the gene patterns using Monocle introduces a spurious spike (at *t *= 10) in the developmental marker *AVP* in healthy cells (Additional file 1: Fig. S6d). In contrast, the gene patterns obtained with Monocle and Slinghsot on the Decipher space are biologically coherent (Additional file 1: Fig. S6f, i). As such, using a dimensionality reduction method like Decipher, which preserves the global ordering of cell states and minimizes distortion, is essential for Monocle and Slingshot to recover biologically meaningful trajectories.

Finally, PAGA loses the global ordering of cell states on both its default space and on the Decipher space (Additional file 1: Fig. S6a, b). The loss is expected because PAGA pre-processes its input space by building a graph using local distances, which does not preserve global cell-state ordering even if the input does. Thus, using Decipher as a dimensionality reduction method, which preserves global ordering and minimizes distortion, is essential for accurate trajectory inference.

With the gene patterns computed by Decipher, we can estimate when transcription factors (TFs) peak along each trajectory, and shed light on the regulatory mechanisms underlying disease derailment. By computing this for all TFs, we found that TFs are upregulated in concert at specific locations along normal hematopoiesis (Additional file 1: Fig. S7a). In contrast, all AML patients display a global loss of TF coordination, including the peak at blast0, which is lost in AML precisely when *NPM1* mutations appear (Additional file 1: Fig. S7a, b). At the level of individual TFs, we confirmed the known upregulation of the homeobox genes (*HOXA9 *[73], *HOXB2*) and their cofactors *MEIS1 *[74] and *PBX3 *[74], as well as the downregulation of *GATA1 *[75] when *NPM1* mutations appear (Fig. 5c). However, we also found diverse TF expression dynamics (e.g., *LCOR*, *AHR*), illustrating that summarizing by peak expression is insufficient to capture the complexity of transcriptional regulation (Fig. 5c). We thus developed a more systematic approach to quantify altered expression between two trajectories next.

### Basis decomposition reveals specific gene dysregulation in AML

**Fig. 6:**
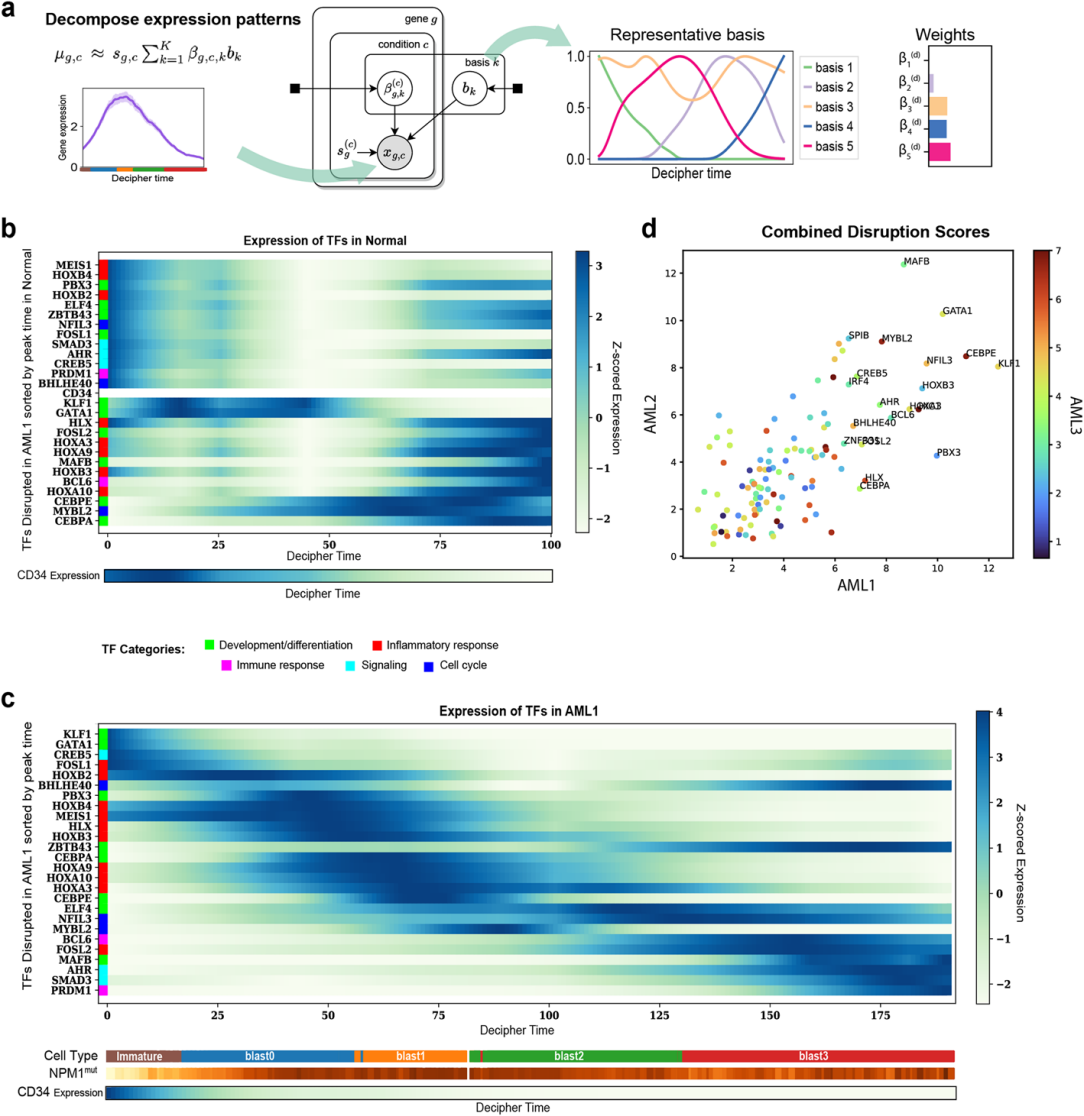
Decipher components unlock transcription factor dynamics. **a** Overview of Decipher’s probabilistic basis decomposition and disruption quantification method. The neural basis decomposition learns the dominant representative patterns and decomposes each gene expression pattern onto them; the coefficients on the basis for each gene are compared between the normal branch and the AML branch to compute the disruption score (Methods section). **b**, **c** Timing of TF expression in normal (**b**) and AML1 (**c**) samples. Heatmaps show log-transformed and z-scored expressions for the top 20 TFs with the highest combined disruption scores in AML1 and known TFs from the literature, sorted by timing of maximum expression in AML1. The HSPC marker *CD34 *[74, 77] is included for calibration. Colorbars correspond to cell type and proportion of NPM1-mutated cells among the 30 nearest neighbors of each cell in AML1; both are smoothed over the 50 nearest neighbors in the Decipher space. **d** The combined disruption score (Methods section) of TFs in three AML patients, with the x-axis representing disruption scores in AML1, the y-axis representing disruption scores in AM2, and the point color representing disruption scores in AML3. Labeled points indicate the TFs that are among the top 20 disrupted TFs in at least one patient

To quantify the differences between gene trends along two trajectories, we devised a probabilistic framework that assumes the expression of each gene can be approximated by a weighted combination of a few representative patterns with distinct temporal dynamics, such as ascending, descending, or peaking in intermediate states (Fig. 6a and Methods section). The model further assumes that representative patterns are shared between normal and perturbed trajectories but with a different scale parameter and weights. The representative patterns are mathematically defined as basis functions that are simultaneously inferred with the decomposition parameters and capture dominant dynamics along trajectories. The decomposition weights ($\beta$) indicate which patterns are associated with each gene, and the scale parameter (*s*) indicates the magnitude of expression (high or low) of the pattern. Specifically, we modeled the patterns using Gaussian processes adapted from [76] and approximated them using neural networks (Methods section).

Using the basis decomposition, we can distinguish changes in the temporal dynamics of genes from changes in the overall scale of expression. The *shape disruption* measures the distance between decomposition weights independent of the scale, while *combined disruption* considers both weights and scale (Methods section). We computed shape and combined disruption scores for all genes and identified conserved (unchanged between normal and perturbed) and disrupted (altered) genes in both measures (Additional file 1: Fig. S8a). For instance, homeobox genes *HHEX* and *HOXB2* have a high shape disruption score (Additional file 1: Fig. S8b). Inflammatory genes *CXCL3* and *CXCL8*, as well as *KMT2A* (which interacts with Menin, a target of *NPM1*-mutated AML clinical trials [78]), have a high combined disruption score, as they are upregulated in immature AML cells compared to normal, with substantial differences in scale (Additional file 1: Fig. S8a, c).

Many of the genes with the highest shape disruption (e.g., *HHEX*, *HOXB2*, Additional file 1: Fig. S8b) arise early in AML, around the initiation of *NPM1* mutation, and subsequently drop to similar expression levels in advanced blasts, highlighting the importance of enriching our data with early leukemic progenitors to detect such initial events. Others, such as the TNF$\alpha$ pathway (*CCL4*, *PHLDA1*) and the oxidative phosphorylation (*ALAS1*, *TCIRG1*) genes, peak late and offer insight into the final transitions to disease (Additional file 1: Fig. S8d, e).

The ability of disruption scores to associate genes with cell-state transitions offers an opportunity to understand regulatory events underlying derailment in AML. We collated a list of TFs with high disruption scores and known TFs from the literature and found that they are transcribed in coordinated waves in normal hematopoiesis and as a cascade in AML (Fig. 6b, c). The key myelopoiesis regulators GATA1 and KLF1 are at the top of this cascade, followed by *HOXA9* and *MEIS1* (known to be altered specifically in NPM1-mutated AML [74]) at the time *NPM1* appears (Fig. 6c). This suggests that **TET2** mutation disrupts the function of key hematopoiesis TFs, which propagate to additional TFs in the gene regulatory network. Interestingly, the upregulation of HOX TFs upon *NPM1* mutation coincides with an increase in interferon type 1 signature genes [71, 79] including *LY6E*, *FAM46C*, *ADAR*, and *TMEM238* (Additional file 1: Fig. S9a). This is in contrast to components of type II interferon (IFNg) response, including MHC-II genes that are upregulated most in early immature cells (Additional file 1: Fig. S9b), suggesting their link to *TET2* mutation. Moreover, we observe that genes in the proinflammatory TNF$\alpha$ pathway (Additional file 1: Fig. S8c), the inflammatory cytokine gene *IL-1*, and AP-1 component *FOS* (Additional file 1: Fig. S9c) are upregulated towards the end of the trajectory in the transformation to blasts.

To determine whether dysregulated TF patterns can generalize, we compared the top disrupted TFs of all 3 AML patients (Additional file 1: Fig. S10) and observed strong similarity in the combined disruption score across patients, with key TF genes including *HOXB3*, *HOXA3*, and *GATA1* among the top 20 disrupted TFs in AML1 and AML2 (Figs. 6c, d and S10a). AML3 presents other disrupted regulators, such as *MYC*, which is known to be overexpressed in AML [80] (Additional file 1: Fig. S10b). In all 3 patients, we observe a cascading effect of key disrupted TFs over time (Additional file 1: Fig. S10a, b) that is further supported by single-cell ATAC-seq data (Additional file 1: Supplementary Information). Our approach thus resolves the timing of TF activity concerning significant events such as genetic mutations and signaling pathway activation, guiding further studies of regulatory relationships.

To evaluate our disruption scores in the context of a larger cohort, we performed differential gene expression analysis between *NPM1*^mut^ AML and normal samples using a publicly available cohort of 125 *NPM1*^mut^ AML samples and 16 HSC-enriched normal subpopulations (see Data availability; Methods section). Examining the top 20 disrupted TFs in AML1 (combined disruption score), we found that 18 out of 20 are differentially expressed (absolute log2 fold-change > 1.16; $p<$ 6.39e−03) in the larger cohort. We also find 12 out of the top 20 disrupted TFs in AML2 (absolute log2 fold-change > 1.6; $p<$ 4.06e−03) and 7 out of the top 20 TFs in AML3 (absolute log2 fold-change > 1.52; $p<$ 6.01e−09) to be differentially expressed in bulk data. Interestingly, the TFs that are not detected in the larger cohort (e.g., *POU2AF1*, *MAFF*; Additional file 1: Fig. S8f) exhibit altered expression in early immature cells whose signal would be diluted in bulk data dominated by blasts. It is noteworthy, however, that TFs disrupted in AML1 that overlap with the bulk differentially expressed are not the most enriched genes but rather among the top 26% of genes ranked according to absolute log2 fold-change. For example, six *HOX* family genes (Fig. 6b, c) have an average rank of 14,376 out of 48,850 genes in the bulk analysis. The most differentially expressed genes reflect enrichment in terminal blasts, whereas our profiling of early immature cells and computational modeling of their dynamic gene expression deciphers regulators of leukemic initiation.

### Comparative analysis of early-occurring epigenetic mutations in AML

We applied Decipher to compare derailment mechanisms between *TET2*^mut^ and *DNMT3A*-mutated patients. *TET2* and *DNMT3A* are epigenetic regulators with opposing roles—*DNMT3A* adds, and *TET2* removes methyl groups [81]. Mutations in these genes, along with ASXL1, are observed in pre-leukemic lesions and clonal hematopoiesis, supporting a stepwise mechanism for AML progression by which normal HSPCs acquire mutations in epigenetic modifiers before a transformative event such as an *NPM1* driver mutation [59]. We compared derailment in these epigenetic contexts to investigate how disease-priming mutations with opposing roles lead to similar vulnerabilities and to determine whether they share mechanisms of leukemogenesis.

We thus profiled unsorted and CD34-sorted bone marrow cells from *DNMT3A*^mut^ patients (AML13–17), three of whom also harbor *NPM1* mutations (Additional file 3: Table 3), and used our original *TET2*^mut^ cohort to annotate the maturation stages of clusters in this single-cell data (Methods section). In the *DNMT3A*^mut^ patients, Decipher also successfully aligns AML maturation and normal HSPC differentiation along the Decipher 1 axis and resolves disease derailment along Decipher 2 (Additional file 1: Fig. S11a–d). We found that in the two *DNMT3A*^mut^ *NPM1*^mut^ patients with sufficient sorted cells (AML14, AML15), PROM1 marks blast0 states (Additional file 1: Fig. S11b, d), and 8 of the top 20 disrupted TFs overlap, including regulators of myeloid lineage commitment and differentiation (*CEBPE*, *HOXB3*, *AHR*, *KLF2*, *MYBL2*), inflammatory response (*JUND*), homeobox cofactor (*MEIS1*), and *ZBTB20*. Interestingly, the reduction in oxidative phosphorylation pathway is enriched along Decipher 2 in both *DNMT3A*^mut^ and *TET2*^mut^ patients (Additional file 3: Table 4).

For a more comprehensive comparison of epigenetic mutations, we identified disrupted TFs according to both shape and combined disruption (Additional file 1: Fig. S11e). Many combined disrupted genes partially overlap; for example, *CEBPE* and *HOXB3* are disrupted in both *TET2*^mut^ and *DNMT3A*^mut^ patients, while interferon regulator *IRF8* shows higher combined disruption in *TET2*^mut^ and inflammatory response regulator *JUND* shows higher shape disruption in *DNMT3A*^mut^ [82, 83]. Decipher thus provides a framework for the unbiased characterization of patient-specific disease trajectories and for the comparative analysis of disease mechanisms between patients and genetic backgrounds.

### Decipher characterizes disease onset in gastric cancer cohorts

**Fig. 7:**
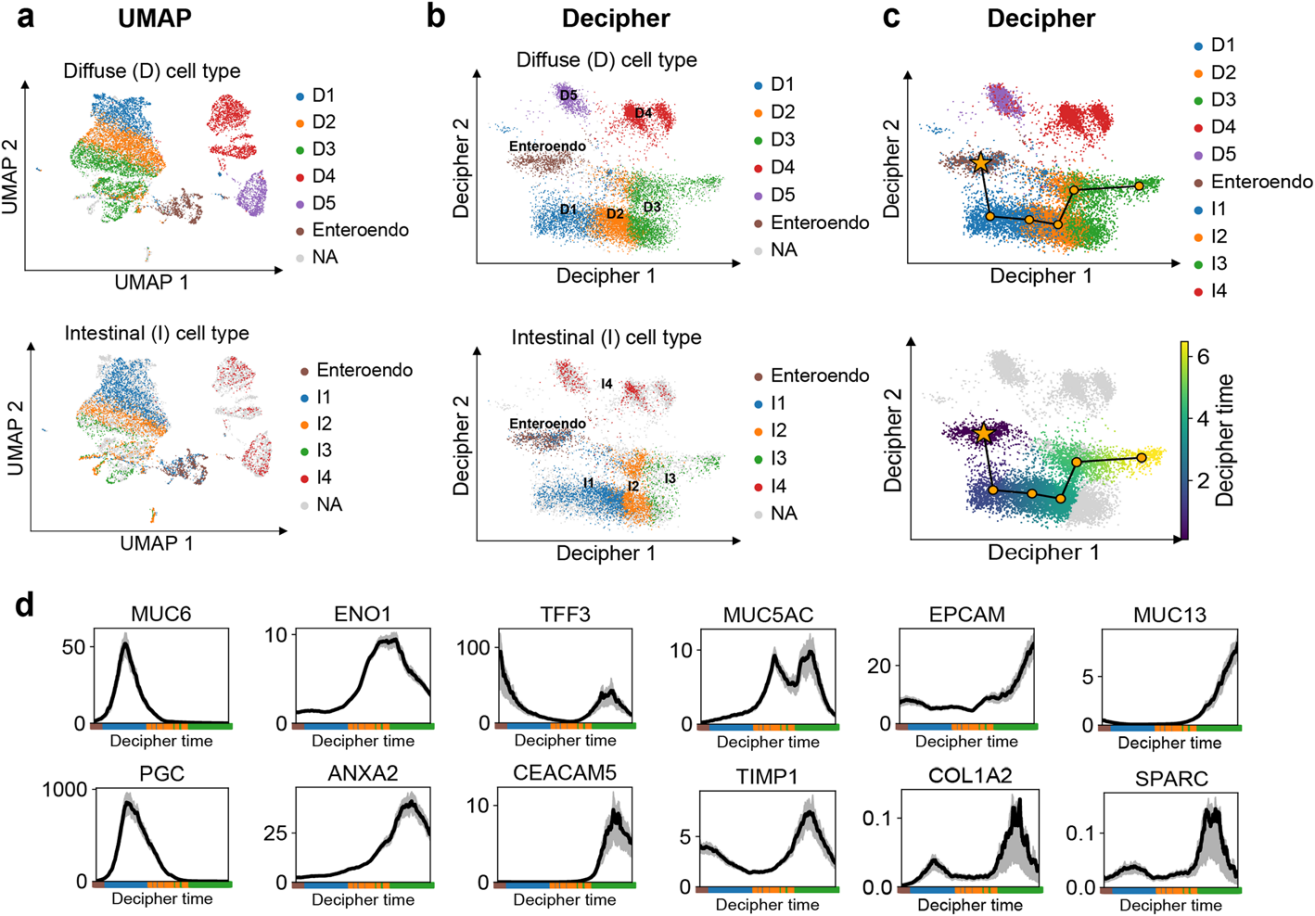
Decipher aligns gastric cancer onset. **a** 2D UMAP projection and trajectory inference with Monocle applied to scRNA-seq data from 24 gastric cancer (GC) and precancerous lesions [84]. **b–c** Decipher reveals the order of cancer stages while still harmonizing the different types of cancer without requiring a batch correction method or a dimensionality reduction method for visualization. We plot D (top) and I (bottom) cells separately, but Decipher is trained on all cells. Cells in Decipher space are colored by cancer progression stages in DGC and IGC (**b**), and the inferred Decipher trajectory and Decipher time on merged data are shown in **c**. **d** Decipher’s reconstructed gene patterns for known markers of progression states along the shared trajectory

In addition to pairwise comparisons, Decipher can be applied to study early and stepwise transitions in large disease cohorts. To illustrate this, we analyzed scRNA-seq data from intestinal (IGC) and diffuse (DGC) primary gastric tumors and paired adjacent non-malignant tissue from 24 patients [84] (Fig. 7a). Each type of gastric cancer was previously shown to capture a partial disease trajectory—specifically, cell-state transitions I1 to I3 in IGC, and D1 to D3 in DGC [84]. However, visualizing the data of all patients with UMAP suggests alternate cell-state transitions that are incorrect; for example, it suggests that enteroendocrine (non-malignant) cells transition to I3/D3 states before I2/D2 and I1/D1 (Fig. 7a and ref. [84]).

We pooled all patients together and used Decipher to derive a more interpretable representation of tumor progression in the two GC types. The Decipher 1 axis correctly aligns cells along a continuous shared progression, with non-malignant enteroendocrine, I1 and D1 cells at the start, followed by I2 and D2 intermediate states, and finally I3 and D3 malignant states (Fig. 7b). The inferred Decipher trajectory (Fig. 7c) reveals the upregulation of malignancy-associated genes at the correct cell-states, including *MUC6* and *PGC* in normal gland mucous cells, *ENO1* in intermediate state I2, and *MUC13* and *CEACAM5* in malignant states (Fig. 7d). The alignment of GC types also illuminates the relative timing of disease progression, predicting that premalignant cells transform to malignancy in IGC later than in DGC, as I1 extends further than D1 along Decipher 1 and Decipher time. The Decipher 2 axis, on the other hand, separates the diffuse-like malignant state (D4) in DGC, illustrating the drastic derailment from D3 upon upregulation of key DGC markers such as *COL1A2* in D4. Finally, fibroblasts and endothelial cell states (I4, D5), which are not a part of the progression of cancer, are preserved as distinct states, highlighting Decipher’s ability to represent both continuums and distinct states.

## Discussion

Decipher is a deep generative model designed to learn and visualize joint representations of normal and perturbed data. Unlike single-cell data analysis approaches that carry out latent factorization and 2D projection as distinct steps, Decipher uniquely merges the two within a single probabilistic, hierarchical structure. As a result, Decipher not only provides direct 2D visualization but also captures more intricate information while remaining interpretable and discovering dependencies between the underlying latent factors. In addition to visualizing cell states with less distortion than other methods, Decipher can use the joint representation to infer trajectories of cell-state transitions and identify genes with disrupted expression patterns using a novel basis decomposition technique. Decipher scales to large cell numbers due to its VAE model formulation, which allows for stochastic variational inference [85]. Other approaches deploy VAEs [37, 42] or Gaussian process latent variable models (GP-LVMs) [86, 87] for nonlinear dimensionality reduction. Still, neither are capable of simultaneous 2D visualization, and GP-LVMs do not scale to large datasets.

In simulated data, Decipher preserves sparsely sampled cell-state trajectories and maintains the geometry of the data better than other methods. We anticipate that Decipher will be a valuable tool for discovering how perturbation or disease initiations derail development. It successfully separates normal and mutant cell trajectories in a mouse model of PDAC bearing a mutation in the tumorigenic driver Kras, revealing the activation of distinct molecular pathways in response to oncogenic stress. Decipher’s broad applicability is also evinced by its successful joint mapping of transitions from premalignant to malignant cell states in two subtypes of gastric cancer.

The early stages of tumor initiation are understudied in primary AML, and findings from animal models only partially translate to humans [88]. AML presents significant genomic and transcriptomic heterogeneity, suggesting multiple vulnerable states and origins of derailment from normal hematopoiesis [20, 89]. Decipher is able to characterize patient-specific divergence from normal myeloid differentiation, confirmed by NPM1 genotyping, whereas other integration methods distort the global geometry of trajectories. Our work discovered and characterized a rare subset of *PROM1*+ cells in NPM1-mutated samples that likely define a pre-leukemic cell population [90, 91]. Decipher also revealed that *NPM1* mutations trigger the upregulation of inflammatory genes and IFN responses following the loss of coordinated myeloid TF expression due to *TET2* mutations. These findings are consistent with studies linking high *HOX* expression to mutant *NPM1* and its aberrant cytoplasmic localization in leukemic persistence [92, 93].

Recent studies in mice demonstrate that loss of *Tet2* induces the expansion of aberrant inflammatory monocytic populations by establishing a pro-inflammatory microenvironment [71, 94]. Similarly, we find the upregulation of IFN type 2 (specifically, MHC-II) genes in early *TET2*-mutated cells in primary samples. *NPM1* has been reported to regulate IFNg-inducible genes in HeLa cells [95], but the link is not established in AML. Our pseudo-time-resolved characterization of transcriptional dynamics shows genes involved in IFN type 1 response to be highly expressed, specifically in transition to aberrant NPM1-mutated progenitor cells, coinciding with the expression of *HOX* TF genes. In further transformation to blasts, we observe the upregulation of genes encoding TNF$\alpha$, IL-1, and FOS. The diverse patterns of chemokines and cytokines along the leukemic transformation trajectory also point to possible dysregulated interactions among them [96]. Inflammatory cytokines such as IL1 can indeed regulate hematopoietic stem cells and promote disease progression in models [97]. While our data does not contain significant non-leukemic myeloid populations and cannot resolve the cellular source of the IL6 and other cytokines responsible for inducing these programs, their stark upregulation along the Decipher 2 component supports work in *Tet2* murine models [73], suggesting this inflammation drives cellular plasticity enabling leukemogenesis, rather than merely being coincident to it [67, 73–76].

In addition to its role in shaping the AML microenvironment [94], cell-intrinsic inflammation is induced by *NPM1* perturbation in mice, leading to myelodysplastic syndrome-like phenotypes [98] and driving progression to AML [99]. These observations motivate future studies on inflammatory response due to *NPM1* perturbation, compared to epigenetic remodeling in clonal hematopoiesis, and studies disentangling the role of pre-existing epigenetic mutations in inducing an inflammatory environment crucial for disease transformation. Extending the application of Decipher to other primary cancer samples as well as animal models can guide therapeutic strategies for modulating the TME and cell-intrinsic effects by attenuating the inflammatory response and, in turn, inhibiting cancer progression or increasing sensitivity to treatments. As a method, Decipher could also be extended in multiple ways: (i) the Decipher space could be leveraged for transferring cell-type annotations [43, 100], (ii) the complementarity between trajectory inference methods and the Decipher space could be further studied with other trajectory inference methods [101, 102], and (iii) Decipher could be extended to characterize multimodal datasets more effectively.

## Conclusions

In conclusion, Decipher successfully integrates data across samples and disease contexts. It aligns cell states in a novel 2D visualization space with greater faithfulness than current state-of-the-art dimensionality reduction tools. Decipher’s VAE architecture additionally allows for the construction of gene trends along any specified trajectories, with the comparison of these trends facilitated by a novel basis decomposition method.

## Methods

### Decipher

Given a dataset of single-cell gene expression $(x_{i,g})$ of *N* cells and *G* genes, Decipher models the expression of genes in cells by learning multiple hidden representations of each cell, at increasingly finer detail: the Decipher components *v* gives a high-level two-dimensional representations, and the ten-dimensional Decipher latent factors *z* are more refined representations of cells. To do so, Decipher is a generative model that extends traditional variational autoencoders—such as scVI and related models [37, 45]—by adding a higher-level two-dimensional latent space on top of their standard latent space. This extra layer allows Decipher to model more complex cell state distributions with latent factors that are potentially dependent. At the same time, the top-level two-dimensional latent variables provide a ready-to-use visualization of the cellular landscape, eliminating the need for separate dimensionality reduction techniques. Because Decipher models gene expression and the visualization layer together, it also enables practitioners to compute gene expression patterns along any trajectory or region in the two-dimensional visualization.

The Python code implementing our method is available at https://github.com/azizilab/decipher. For each method presented below, we reference its corresponding Python function. Our Python code follows the architecture of the scanpy package [103], with computation functions in a .tl submodule and the plotting functions in a .pl submodule. In the code snippets of the methods below, we assume that we have imported the decipher package as follows import decipher as dc and that the data of interest is in an AnnData object called adata. For instance, training Decipher is done with dc.tl.decipher_train(adata) and plotting the Decipher space colored by cell type is performed with dc.pl.decipher(adata, color="cell_type"). The Decipher model is also implemented in the scvi-tools package [104].

#### The generative model

Decipher begins by representing each cell *i* with a two-dimensional standard normal latent variable $v_i$, termed *Decipher components*. The Decipher components represent the two principal axes of cell heterogeneity, such as cell type variation or stages of disease progression. They directly serve as a two-dimensional visualization.

A learnable neural network *f*—the first *decoder*—maps each $v_i$ to a distribution over medium-dimensional vectors $z_i$, representing cell states. Each $z_i$ is sampled conditionally on $v_i$ from the distribution, and we refer to $z_i$ as the *Decipher latent factors*. The $z_i$ are medium-dimensional; they contain richer information about cell *i* than $v_i$, but are still substantially lower dimensional than the number of genes (we set the dimension to 10 in our experiments). The latent factors are comparable to those of other VAE-based or matrix-factorization-based methods [37, 105, 106].

A second neural network *h*—the second decoder—maps $z_i$ to normalized gene expression means $(\mu _{i,g})_{1\le g\le G} = h(z_i)$ for each gene *g* in cell *i*. The output layer of *h* uses a softmax activation to ensure normalized expression across genes. The final observed counts $x_{ig}$ are generated from a negative binomial distribution with mean $\mu _{i,g} \cdot l_i$, where $l_i$ is the observed library size of cell *i*, and with dispersion $\theta _g$, which are learned for each gene *g*.

This generative process is represented in Fig. 1b and is described mathematically as follows:1

The mapping functions *f* and *h* are neural networks. In practice, Decipher uses a single linear layer for *h* to limit distortion [44]. *f* has two linear layers interleaved with ReLU activations. The last layer of *f* produces a vector in $\mathbb {R}^{2L}$ that is split to form two outputs: $f_{\texttt {mean}} \in \mathbb {R}^L$ and $f_{\texttt {var}} \in \mathbb {R}^L$. Other distributions can replace the choice of negative binomial distribution if the user believes it is more appropriate for the data at hand.

##### High-level summary

The Decipher components $v_i$ represent the high-level organization of the cells and form the Decipher space. This space provides a ready-to-use 2D representation of the data without requiring further projection methods such as UMAP or t-SNE. It offers direct visual access inside the probabilistic model. Then, through the neural network *f*, each $v_i$ induces a cell state $z_i$, a more detailed representation of cell *i*. The space of the $z_i$ corresponds to the latent space of traditional variational-autoencoders.

#### Decipher’s inference

Given observed gene expression data $\mathcal {D} = \{x_{i,g}\}^{1\le i \le N}_{1\le g \le G}$ (if the data comes from multiple patients or samples, the observations are simply concatenated), and parameters $\{(\theta _g)_{1\le g \le G }, f, h\}$, Decipher’s probabilistic model defines a posterior $p\left( v, z \mid \mathcal {D} \right)$ over the latent variables $v = (v_i)_{1\le i \le N}$ and $z = (z_i)_{1\le i \le N}$. We approximate this exact posterior with a variational approximation $q\left( v, z \right)$ learned with variational inference [107–109].

We use amortization over the local variables $v_i, z_i$ as a function of the observations $x_i$. The variational family becomes $q(v, z) = \prod _{i=1}^N q(v_i , z_i | x_i)$ which always factorizes as

$$\begin{aligned} q(v, z) = \prod _{i=1}^N q(v_i | z_i, x_i)q(z_i | x_i). \end{aligned}$$

The amortized distributions are set to diagonal Gaussian distributions with parameters (mean and variance) given by neural networks—the two encoders. The first neural network transforms $x_i$ to the mean and the variance of the distribution $q( z_i | x_i )$, and the second neural network transforms $(z_i, x_i)$ to the mean and the variance of the distribution $q(v_i | z_i, x_i)$. We denote them as $d^{\rightarrow z}_{\texttt {mean}}(x), d^{\rightarrow z}_{\texttt {var}}(x), d^{\rightarrow v}_{\texttt {mean}}(x,z),$ and $d^{\rightarrow v}_{\texttt {var}}(x,z)$, such that:

$$\begin{aligned} q(z_i | x_i) = \mathcal {N}(d^{\rightarrow z}_{\texttt {mean}}(x_i), d^{\rightarrow z}_{\texttt {var}}(x_i)), \quad \text {and} \quad q(v_i | z_i, x_i) = \mathcal {N}(d^{\rightarrow v}_{\texttt {mean}}(x_i,z_i),d^{\rightarrow v}_{\texttt {var}}(x_i,z_i)). \end{aligned}$$

Variational inference seeks to minimize the KL divergence between the variational posterior *q* and the exact posterior $p( ~\cdot \mid \mathcal {D})$. It is equivalent to maximizing a lower bound of the evidence, called the ELBO [109]:

$$\begin{aligned} ELBO(q) = \sum \limits _{i=1}^N \mathbb {E}[q(v_i,z_i)]{ \sum \limits _g \log p(x_{i,g}|z_i, \theta _g) + \log \frac{p(z_i |v_i)}{q(z_i|x_i)} + \beta \log \frac{p(v_i)}{q(v_i|z_i, x_i)}}, \end{aligned}$$

where $\beta$ is a scalar controlling the importance of the prior $p(v_i)$, between 0 (no prior) and 1 (standard ELBO) [110].

Because we chose the variational posteriors *q* to be Gaussian distributions, we can reparameterize the expectations to sample unbiased low-variance estimates of the ELBO. To obtain a sample for $(z_i,v_i)$ from $q(v_i, z_i | x_i ) = q(v_i | z_i, x_i) q(z_i | x_i)$, we first sample a reparameterized $z_i$ from $q(z_i | x_i)$ and then sample a reparameterized $v_i$ from $q(v_i | z_i, x_i)$ [111].

The gradients are then computed using automatic differentiation, to update all the parameters: $\theta _g$, the decoder neural networks *f* and *g*, and the encoder neural networks $d^{\rightarrow z}, d^{\rightarrow v}$. To scale up to large datasets of cells, we further subsample the outer sum using a minibatch size of 64 observations to perform stochastic variational inference [85]. We use the Adam optimization algorithm [112] to execute the gradient updates. The code is implemented in Python using Pyro [113].

Because we have little prior on the distribution of the Decipher components *v* (remember that a limitation of other methods is that the prior enforces unrealistic independence between latent variables), we set $\beta$ to a low value $1e-1$ in our experiments.

The Decipher model can be fitted using the function dc.tl.decipher_train(adata).

##### Generating the Decipher space *v* and the latent space *z*

Once the inference is performed, the “encoders” $d^{\rightarrow z}_{\texttt {mean}}(x)$ and $d^{\rightarrow v}_{\texttt {mean}}(x,z)$ give the posterior expected values of $v_i$ and $z_i$ given each cell $x_i$. For each cell $x_i$, we compute (as in any auto-encoder architecture): $\hat{z}_i = d^{\rightarrow z}_{\texttt {mean}}(x_i)$ and $\hat{v}_i = d^{\rightarrow v}_{\texttt {mean}}(x_i, z_i)$.

The Decipher space *v* and latent *z* are automatically computed when calling dc.tl.decipher_train. They are stored in adata.obs["decipher_v"] and adata.obs["decipher_z"].

##### Rotating and aligning the Decipher space

Decipher does not use sample or batch IDs when learning the latent variables, the encoders and the decoders. However, in a post-processing step, the sample IDs (or other annotations) can be optionally used to align Decipher components to represent the most shared and most distinct information between the samples (e.g., perturbed and normal conditions), thus facilitating downstream analysis. This is accomplished by rotating or flipping the *v* components. Like most auto-encoder models (e.g., scVI [37]), the axes of the latent spaces *v* and *z* can be rotated or flipped without changing the likelihood of the data. To automatically rotate and flip the Decipher components given the user preferences, the user can specify if some cell labels should be aligned with a given component. For example, in our analysis, we choose to align the cells’ sample labels (Healthy and AML) along Decipher 2 and the cells’ cell-type labels (ordered from blast0 to blast3) along Decipher 1.

Given cell labels and their target alignment axis (e.g., ordered cell types along Decipher 1, ordered cell sample IDs along Decipher 2), we try 100 rotations (from 0 to $2\pi$) and all possible axis flips (2 for $v_1$, 2 for $v_2$), and pick the setting that maximizes the correlations between the cell labels and their target Decipher axis.

This is accomplished by calling the dc.tl.decipher_rotate_space function.

#### Construction of trajectories and gene patterns

The Decipher components $(v_i)$ organize cells along visual trajectories. For instance, there are two trajectories in the joint AML-healthy data: one for healthy maturation and one for AML progression (Fig. 4c). The trajectories could be traced manually by the user or obtained by trajectory inference methods. Still, we propose a simple automated determination of the trajectories using, as input, the marker genes for the beginning and the end of the trajectories.

Given the cell representations $(v_i)$ in the Decipher space and $(z_i)$ in the latent space, we first cluster cells using the Leiden algorithm [114] on the latent representations $(z_i)$—we use the representation $(z_i)$ to cluster the cells because they contain more detailed information about the cells than the $(v_i)$ (Fig. 5b, left). We then compute a minimum spanning tree between the clusters’ centroids using the distances in the Decipher space—we use the distance in Decipher space because the high-level geometry of the data is better captured by the $(v_i)$ (Fig. 5b, middle left). Finally, we use the provided marker genes to identify the trajectories’ beginning and end, from which we compute the shortest path in the minimum spanning tree (Fig. 5b, middle right). We use linear interpolation to form parameterized trajectories $\gamma : t \mapsto v(t)$ in the Decipher space (Fig. 5b, right). The time *t* that parametrizes the trajectories is called the *Decipher time*, and we compute one trajectory per sample in our analysis ($\gamma _{\text {AML}}$ and $\gamma _{\text {healthy}}$). If the analysis requires it, more trajectories or less could be computed.

The procedure is described in Algorithm 1 and is visually represented in extended data (Fig. 4a). The trajectories are computed using the function dc.tl.trajectories.

**Figure:**
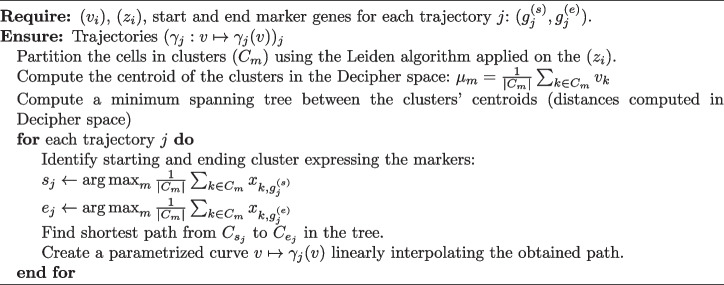
**Algorithm 1** Trajectory paths construction with Decipher

##### Trajectory alignment using Decipher time

While trajectory alignment approaches, e.g., with dynamic time warping [115, 116], can alter the relative lengths of trajectories by locally compressing or stretching them and potentially missing rare cell states, our jointly inferred Decipher time obtains a common time axis for both trajectories. Decipher assigns a pseudo-time value to each location along the trajectories, called the Decipher time. This is determined by calculating the curvilinear coordinate *t* along each trajectory within the Decipher space. To assign a Decipher time to any cell from our data, we project the cells onto the trajectories. That is, we compute for each cell *i* in each sample *j* (healthy or AML), the closest point $\gamma _j(t^*)$ on the dataset-specific trajectory ($\gamma _{\text {healthy}}$ or $\gamma _{\text {AML}}$), and assign the time of this trajectory point $t^*$ to cell *i* (Fig. 1c). The Decipher time is computed using the function dc.tl.decipher_time.

##### Reconstruction of gene expression by Decipher

Given a trajectory $\gamma$ of Decipher components, we can obtain the gene expression along this trajectory using the decoder neural networks in the Decipher probabilistic model. Indeed, we recall that the encoders and the decoders in the Decipher model can convert any gene expression into Decipher components and vice versa. Mathematically, given some Decipher components on the trajectory $v:= \gamma (t)$, we use the decoders to compute the associated expected latent factors $z:= f_{\texttt {mean}}(v)$, followed by the expected normalized gene expressions $\mu := h(z)$, that can further be scaled up to the desired library size by multiplying it by $\ell$. Because Decipher is a probabilistic model, it is also possible to obtain probabilistic gene expression samples instead of a single estimate. This is particularly useful to obtain some model uncertainty around the reconstructed gene expression (Fig. 5a). To achieve this, we sample multiple latent factors given the Decipher components as $z_m \sim \mathcal {N}\left( {f_{\texttt {mean}}(v), f_{\texttt {var}}(v)}\right)$ (instead of just $f_{\texttt {mean}}(v)$) and we compute their expected gene expression $\mu _m = h(z_m)$.

The Decipher gene expression patterns are computed using the function dc.tl.gene_patterns.

#### Basis decomposition

To quantify the difference in patterns between two trajectories, we need a metric that accounts for the temporal order of cells—two genes may have the same mean expression value but opposite patterns, e.g., ascending versus descending along trajectories. Existing methods that encode temporal dependencies are limited in modeling assumptions and scalability. For instance, tradeSeq [117] performs trajectory-based differential expression; however, approximating gene patterns with splines may not be appropriate for complex transcriptional programs, e.g., with cascades of mutations leading to cancer. Additionally, relying on built-in denoising limits compatibility with preprocessed data (e.g., from VAEs). Methods such as DPGP [118] utilize Gaussian processes model distributions of all functions over time; however, they are computationally expensive. This motivates a metric that not only accounts for the order of cell states and uncertainty but is generalizable and scalable to the size of standard single-cell datasets. For this, we develop a method that decomposes every gene pattern on a basis of dominant patterns learned with neural networks. Comparing different patterns then becomes a comparison of their basis weights.

##### The data

The input data for the basis decomposition method is the collection of gene patterns observed over a time or pseudotime axis in different conditions, such as normal, disease, or perturbed (Fig. 5a). Similar to the trajectory $\phi : t \mapsto \phi (t) =: v$ that maps pseudo-time to Decipher components, we define a gene pattern $\mu _{g,c}$ for a gene *g* under condition *c* to be the function $t \mapsto \mu _{g,c}(t)$ that represents the expected expression of gene *g* at time *t* in condition *c*. With Decipher, these can be the gene expression patterns reconstructed from the inferred trajectories. In a more general setting, these patterns can be obtained with time-series bulk RNA-seq or trajectory inference methods [119, 120], applied in advance to single-cell RNA-seq data, or other dynamic features, such as chromatin accessibility (measured with ATAC-seq) or protein expression (CITE-seq).

The set of all genes is *G*, and the set of conditions is *C*. For simplicity of the exposition, we restrict the conditions to $C = \left\{ \text {healthy}, \text {disease}\right\}$. The input data is the collection of functions $\mathcal {D} = \left( \mu _{g,c} \right) _{g \in G, c \in C}$. The data has $|G| \cdot |C|$ observations, each of which is a function. In the proposed probabilistic model, each pattern $t \mapsto \mu _{g,c}(t)$ is considered a single observation.

##### The model

In light of commonly used generative models [121], the proposed model is a linear factor model operating in function space. For gene *g* and condition *c*, the model associates the data point $\mu _{g,c}$ with a latent scalar $s_{g,c}$—the *gene scale*—and a latent vector $\beta _{g,c}$ of *K* dimensions—the *gene shape*. Each dimension *k* corresponds to a latent basis pattern $t \mapsto b_k(t)$. The $\beta _{g,c,k}$ are coefficients for the function $\mu _{g,c}$ in this basis. The coefficient $s_{g,c}$ is the intrinsic scale of gene *c* in condition *g*, which will scale up or down the pattern computed from the bases, which, in contrast, are constrained to be between 0 and 1. The observations $\mu _{g,c}$ and the latent basis $b_k$ are functions. The scale $s_{g,c}$ and the weights $\beta _{g,c,k}$ combine the latent bases $b_k$ to generate the observed function $\mu _{g,c}$. That is, informally, $\mu _{g,c} \approx s_{g,c} \sum \nolimits _{k=1}^K \beta _{g,c,k} b_k$. Each basis function $b_k$ forms a representative pattern shared by multiple genes.

##### The weights $\beta _{g,c,k}$

To ensure the interpretability of the weights, the model mimics methods like mixture or topic models and draws positive weights that sum to 1. With this, a non-zero weight $\beta _{g,c,k}$ signifies that gene *g* in condition *c* exhibits the representative pattern *k*. Specifically, the weights vectors $\beta _{g,c}$ are drawn independently from a Dirichlet distribution with concentration parameter $[\eta , \eta , ..., \eta ]$, denoted as $\text {Dir}(\eta )$, and with density

$$\begin{aligned} p(\beta | \eta ) = \frac{1}{B(\eta )} \prod _{k=1}^K \beta _k^{\eta -1}, \end{aligned}$$

where $B(\eta ) = \Gamma (\eta )^K / \Gamma (K\eta )$ and $\Gamma$ is the Gamma function. In terms of negative log-likelihood, this prior induces a regularization of the coefficients that will lead to sparse $\beta _k$ when $\eta < 1$ and $\beta$ closer to $\frac{1}{K}$ when $\eta>1$. We choose $\eta < 1$ to associate the basis with dominant patterns and thus obtain a better interpretability of the basis.

##### The basis functions $b_k$

To sample the basis functions $b_k$, we represent them as neural networks and sample each $b_k$ by drawing its neural network parameters. Concretely, each basis function is modeled by a one-dimensional neural network with two hidden layers of 32 units each, followed by the tanh activation. The neural network $b_k$ is of the form

$$\begin{aligned} b_k : \mathbb {R} \rightarrow \mathbb {R}^{32} \rightarrow \mathbb {R}^{32} \rightarrow \mathbb {R} \end{aligned}$$

and its parameters are denoted $\phi _k$. Each $\phi _k$ is sampled from a centered diagonal normal distribution, and the variance of each of its coordinates is set to the inverse of the input dimension of the linear layer in which it appears. The study of infinite neural networks in Neal [122] demonstrates that with wide hidden units (here $32 \gg 1$) and such a prior on the parameters, the induced prior in function space is close to a Gaussian process. Gaussian processes are used in other methods [118] but are hardly scalable. Using neural networks for efficient computations solves the problem. The induced prior in function space is denoted by $\varphi$. Finally, to design more interpretability for the gene scale $s_{g,c}$, we normalized the sampled basis by their maximum value so that the maximum value reached by a basis is 1.

##### The gene scales $s_{g,c}$

The gene scales $s_{g,c}$ are learned as variational parameters (no variational distributions), as the gene scales can greatly vary between genes.

##### The observations $\mu _{g,c}$

Finally, the gene pattern $\mu _{g,c}$ is generated from a distribution parameterized by $\sum \nolimits _k \beta _{g,c,k} b_k$ and the scale $s_{g,c}$. More specifically, $\mu _{g,c}$ is sampled from a Gaussian process^1^ with mean $s_{g,c} \cdot \sum \nolimits _k \beta _{g,c,k} b_k$ and with a white Gaussian noise kernel $(x,x') \mapsto \sigma ^2\delta _{x,x'}$ of variance $\sigma ^2$.

The generative process is represented graphically in Fig. 5a and proceeds as follows:

1. For each factor dimension $k = 1, \dots , K$, draw a basis function $b_k$ from the function prior: $b_k \sim \varphi (b_k)$ (that is draw weights $\phi _k$ according to the prior detailed above)
2. For each gene *g*, and condition *c* do:
  - For each factor dimension *k*, draw the basis weight $\beta _{g,c,k} \sim \mathcal {E}(\eta )$
  - Draw the observed function $\mu _{g,c}$ from $\mu _{g,c} \sim \mathcal{G}\mathcal{P}\left( s_{g,c} \cdot \sum \limits _k \beta _{g,c,k} b_k , (x,x')\mapsto \sigma ^2 \delta _{x,x'}\right)$

For simplicity of notations, the $\phi _k$ are grouped in parameter $\phi$, and the $\beta _{g,c,k}$ into parameter $\beta$.

##### The inference

We learn an approximate posterior on the model variables $q(b_k, \beta _{k,g,c}, s_{g,c})$ using variational inference implemented in the Python probabilistic modeling library Pyro [113], with automatic guides.

The basis decomposition is computed using the function dc.tl.basis_decomposition.

##### The disruption scores

From the inferred model parameters, we design multiple disruption scores that inform us of different types of disruptions for the same gene across two conditions $c_1=$ healthy and $c_2=$ disease.

- The scale disruption highlights the difference in gene scale between the two conditions, e.g., a gene that is up-regulated in one of the conditions. It is defined as $|\log s_{g, c_1} - \log s_{g, c_2}|$.
- The shape disruption highlights the difference in gene shape between the two conditions, e.g., a gene that activated later in one of the conditions and earlier in another. It is defined as $|| \beta _{g, c_1} - \beta _{g, c_2}||$.
- The combined disruption is a combination of both disruption scores to capture a general high-level disruption score, including both shape and scale. It is defined as $||\log (s_{g, c_1} \beta _{g, c_1}) - \log (s_{g, c_2} \beta _{g, c_2})||$.

The disruption scores are computed using the function dc.tl.disruption_scores.

### Data generation

#### Simulated data

To evaluate Decipher’s ability to identify cell state evolution trajectories within its latent space, we simulate data with ground-truth trajectories (Fig. 2), fit the Decipher model on this data, and evaluate the quality of the trajectory reconstruction.

We simulate data in several steps:

1. Sample random locations along the 2d *continuous* trajectories of Fig. 2a.
2. Remove some of the locations up to a certain percentage to simulate rare/low-sample cell state transitions: 100% in Fig. 2a, 90% and 95% in Fig. b, and a varying percentage in Fig. c.
3. The remaining locations are denoted $(z_i)$ and are the ground truth cell states.
4. In particular, we consider the first coordinate of $z_i$ to be the pseudotime of the cell, noted $t_i = z_{i,1}$.
5. Randomly perturb the ground truth cell states to simulate noise $\sigma$: $z'_i \sim \mathcal {N}(z_i, \sigma ^2)$.
6. Sample randomly the weights and biases of a neural network *f* with output dimension of size *d* (in our experiments $d=500$). The neural network is used as a random function to nonlinearly transform the cell state into higher dimensional gene expression.
7. Use this neural network to map the ground truth cell states to the synthetic gene expression: $x_i = f(z'_i)$.

With this simulation, we obtain random gene expression data $(x_i)$ that is organized along an underlying continuous trajectory with possible rare transitions.

#### Evaluation metric on simulated data

We evaluate the quality of a latent space $(z'_i)$ using the global preservation metric presented in Chari and Pachter [41]. We present it here briefly. Our simulation provides ground-truth cell states (the $z_i$). We cluster those cell states into 20 clusters using k-means. We denote $C_j$ the indices of cells in cluster *j*. Then, the global preservation metric from Chari and Pachter [41] computes the pairwise distances between each cluster in the ground-truth cell state space $d_{i,j} = \sum \nolimits _{(i,j) \in C_i \times C_j} \Vert z_i - z_j\Vert$, as well as in the new latent space $d'_{i,j} = \sum \nolimits _{(i,j) \in C_i \times C_j} \Vert z'_i - z'_j\Vert$. The global preservation metric is then the average Kendall-tau correlation between the distances to each cluster in ground-truth space vs new space:

$$\begin{aligned} \frac{1}{20}\sum \limits _i \tau ((d_{i,j})_{j=1}^{20}, (d'_{i,j})_{j=1}^{20}). \end{aligned}$$

Higher is better, with a maximum of 1, indicating a perfect correlation of cluster ordering between ground truth and new latent space. The results are presented in Fig. 2c.

#### AML data collection

The TET2$^{mut}$ AML cohort consists of 12 cryopreserved (DMSO) BM AML samples from the *Banque de cellules leucémiques du Québec* (BCLQ) biobank, with 10 patient specimens collected at the time of diagnosis, and two specimens from the same patient, at diagnosis and relapse (Additional file 3: Table 3). The DNMT3A$^{mut}$ AML cohort consists of 5 cryopreserved (DMSO) BM AML patient samples. FAB information for samples was provided by BCLQ (Additional file 3: Table 3). Karyotyping, as well as mutation (variant) calling, was performed via bulk RNA-sequencing as part of the Leucegene project with data deposited on GEO with accession IDs GSE106272, GSE49642, GSE52656, GSE62190, GSE66917, and GSE67039.

All samples, as well as sorted cells, were profiled using 10X Genomics Chromium Single-cell 3′ for scRNA-seq. For scATAC-seq, cells were subjected to 10X Genomics Chromium Single Cell ATAC Reagent Kits User Guide (v1.1 Chemistry). The resulting nuclei suspension was subjected to a transposition reaction for 60 min at 37 °C and then encapsulated in microfluidic droplets using a 10X Chromium instrument following the manufacturer’s instructions with a targeted nuclei recovery of approximately 5000. Barcoded DNA material was cleaned and prepared for sequencing according to the Chromium Single Cell ATAC Reagent Kits User Guide (10X Genomics; CG000168 RevA). Purified libraries were assessed using a Bioanalyzer High-Sensitivity DNA Analysis kit (Agilent) and sequenced on an Illumina HiSeq 2500 (High Output) and NovaSeq platform at approximately 100 million reads per sample (around 5000 nuclei) at MSKCC’s Integrated Genomics Operation Core.

##### Flow cytometry activated cell sorting (FACS)

For immature cell enrichment, FACS-purified CD34+ or PROM+ cells were subjected to single-cell RNA sequencing. Cryopreserved mononuclear cells were thawed into 10 ml of prewarmed FACS buffer (phosphate-buffered saline (PBS) + $2\%$ fetal bovine serum). Cells were pelleted at $300\times G$ for 5 min and washed again with FACS buffer. Cells were then resuspended in FACS buffer containing Human TruStain FcX^™^ (Fc Receptor Blocking Solution; Biolegend #422301) for 15 min at 4 °C. Antibodies against CD34 (Clone 561; FITC Biolegend 343603) and CD133 (clone 7; PE Biolegend 372803) were subsequently added, and cells were stained for an additional 15 min at 4 °C. Cells were then washed twice with 3 ml of FACS buffer and resuspended in FACS buffer with DAPI. Cell sorting was performed on a Sony SH800.

### Data pre-processing and analysis

#### Data pre-processing

Quantification of counts was done with SEQC [2] and 10X Genomics Cellranger Zheng et al. [123]. Counts outputs were loaded into AnnData format using scanpy 1.7.2 [103]. Cells with low library size were filtered out, with the filtering threshold being selected by the knee-point of a histogram of the log10 of the total counts per cell. We obtained a median of 10,504 cells per sample and a median of 5165 molecules per cell after filtering. Data were then normalized by median library size using sc.pp.normalize_per_cell. Doublet detection was performed using DoubletDetection [124], with 25 iterations. scATAC FASTQ files for each sample were preprocessed to a cell-by-peak count matrix through the CellRanger ATAC pipeline [125] with modifications as described in Alonso-Curbelo et al. [54].

#### Annotation of AML TET2 cohort

All cells from unsorted AML samples were considered (Fig. S2a, b). PhenoGraph [5] clustering was run using 100 principal components and 15 nearest neighbors. Annotation of clusters with low counts or high mitochondrial reads was performed by visual analysis of boxplots for the log10 of counts per cluster, as well as the fraction of reads belonging to mitochondrial genes compared to all genes. We further annotated lymphoid and erythroid clusters using scanpy’s dotplot function to visualize key gene markers. We were able to identify these clusters by analyzing the fraction of cells in each cluster expressing key marker genes as well as the mean expression. Cells forming distinct low-count clusters and additional clusters with high mitochondrial fraction, using Phenograph clustering on a per-sample basis, were additionally removed, resulting in a global cohort of 104,116 cells.

To annotate the maturation stages of leukemic blasts, we computed correlations (scipy.stats.pearsonr) between the mean expression of each cluster and bulk gene expression data from sorted HSPCs [126]. The correlation calculation was limited to the 5277 most varying genes, 3475 of which overlapped with bulk data. Non-significant values ($p>0.0005$) were removed (Fig. S2c). To control for cluster size, Shannon diversity (Fig. S2c) was computed for the distribution of patient IDs in subsamples of *N* = 1000 (approximating the median cluster size) cells from each cluster and averaged across 20 iterations. Paired diagnosis-relapse samples (AML9, AML10) (Fig. S2c) annotations were considered together as they are phenotypically very similar (Fig. S2a). Clusters are ordered within cell-type by decreasing diversity (Fig. S2c).

#### Mutation identification and metrics

We implemented a mutation calling protocol in order to identify mutations in NPM1 and DNMT3A. We first sorted and indexed the patient bam files using samtools [127], then reduced the file to the region containing the gene of interest. The NPM1 gene was analyzed from chromosome position 5:171387116-171411137, and the DNMT3a gene was analyzed from chromosome position 2:25230961-25344590. Files were then merged and indexed, then converted to FASTA format. The indexed bam file was then loaded into the Integrative Genomics Viewer [128], and the alignments were visually analyzed for the presence of the mutation. If a mutation is present, a range of 5–20 base pairs are selected for subsequent single-cell analysis (Additional file 2: Table S1). For single-cell annotation of mutations, we used the previously generated FASTA file and the mutated sequence identified for each patient to search for the presence of the mutated sequence in individual cells.

Because the mutation state may be heterozygous (a cell may have both mutant and wild-type labels), most of our subsequent analysis utilizes our defined *mutation proportion* for each cell. Since our detection of mutation is dependent on expression, which is affected by dropouts in scRNA-seq, we compute an average mutation proportion in the neighborhood of each cell. To compute the mutation proportion, we find the 30 nearest neighbors of each cell on the truncated SVD decomposition (100 components) of the normalized data. The mutation proportion for each cell is then $m/(m+w+1e^{-10})$, where $m=$ the number of cells bearing the mutated copy of the gene and $w=$ the number of cells bearing the wild-type copy of the gene among the 30 neighbors. The heterozygous NPM1 mutation is detected in 5–39 % of cells in each of the unsorted samples.

#### Verification of immature cell enrichment in sorted samples

The primary purpose of the cell sorting was to enrich the populations of CD34+ and PROM1+ immature cells in the data (Fig. S2d). To verify that this enrichment was achieved, we first performed a visual analysis of the UMAP computed on the subset of cells in AML1 originating from the unsorted collection process first, and compared it with the UMAP of cells once the sorted cells were included with the unsorted cells. We verified that enrichment of PROM1 and CD34, along with cells with low NPM1 mutation proportion, was achieved in the UMAP (S2e). We also quantified the expression of CD34 and PROM1 in each of four categories: immature and non-immature cells in the unsorted cells only and immature and non-immature cells in both sorted and unsorted cells. All visualization was performed using scanpy [103].

#### Cell type mapping onto the DNMT3A cohort

To extend the annotations from the TET2 cohort to patients in the DNMT3a cohort, we combined the data for all patients in the TET2 cohort, normalizing by median library size and log transforming across the entire cohort. Cells were then grouped based on their prior cell type annotations, and 700 differentially expressed genes were identified per cell type using the T-test version of scanpy’s [103] *rank_genes_groups()* function. Mitochondrial and Ribosomal genes were excluded from the gene sets. Cells in the DNMT3A cohort were also combined across patients, normalized by median library size, and log-transformed. Cells were then split into clusters using PhenoGraph [5], computed using 100 principal components and *k *= 5. We then computed the cluster centroids of the PhenoGraph clusters in the DNMT3a cohort and the cell types of the TET2 cohort by taking the mean across cells in the cluster, limiting to the differentially expressed genes. Pearson correlation (using scipy 1.7.0 [129]) was computed between the centroids of the two cohorts, and each DNMT3A cluster was labeled with the cell type of the TET2 cell type cluster to which it had the greatest correlation coefficient. We can then apply Decipher on these patients, following our standard analysis pipeline (Fig. S10a).

#### Benchmarking

We evaluated the performance of Decipher on simulated data. To further benchmark the performances of Decipher on real data, we define two metrics based on our prior knowledge of AML and compare Decipher to a large spectrum of commonly used methods.

Since we do not have ground-truth trajectory values for the real data, we build metrics on prior knowledge of AML progression, AML marker genes, and our independently curated cell state annotations: immature, blast0, blast1, blast2, and blast3. Among the healthy immature cells, we further use the markers CD34 and MPO to distinguish early cells (CD34+), late cells (MPO+), and intermediary cells. Our metrics are based on the distances between annotated cell states.

- **Ordering score**: We expect the cell states in a latent space to be spatially ordered along the known cell maturation trajectories. For instance, blast1 should be between blast0 and blast2. Given the orders $o_1 =$ [immature, blast0, blast1, blast2, blast3] and $o_2 =$ [early, intermediary, late], we want the total distances between consecutive cell states to be smaller than the distances between non-consecutive cell states. The triangular inequality guarantees that the ratio of these two quantities (the second over the first one) is maximized when the clusters are perfectly aligned in the right order.
  $$\begin{aligned} \text {order}_j = \frac{\sum \nolimits _{|i_1 - i_2|> 1} \text {distance}(o_j[i_1],o_j[i_2])}{\sum \nolimits _{|i_1 - i_2|= 1} \text {distance}(o_j[i_1],o_j[i_2])}. \end{aligned}$$
- **Divergence score**: We expect the AML trajectory to diverge from the healthy trajectory. That is, the immature cells of the AML sample are close to the early immature cells of the healthy sample. But then, the blast3 cells of the AML sample are far from the late immature cells of the healthy sample.
  $$\begin{aligned} \text {divergence}_j = \sum \limits _{\begin{array}{c} c_1 \in o_1\\ c_1 \ne \text {immature} \end{array}}\sum \limits _{\begin{array}{c} c_2 \in o_2\\ c_2 \ne \text {early} \end{array}} \text {distance}(c_1,c_2) - 2*\text {distance}(\text {immature(AML),immature early (healthy)}). \end{aligned}$$
  This metric is higher when non-immature AML cells and non-early healthy cells are far from each other and when the immature AML cells and early healthy cells are close to each other.

These metrics attempt to capture our high-level prior knowledge of AML. They summarize the latent space of each method in two numbers: the ordering score and the divergence score. For further details of each method, one can also directly analyze the visualization of the latent space of each method (Fig. S3).

Below are the benchmarked methods, the implementation we used, the hyperparameters, and which latent space we used to compute the metrics:

- **PCA**
  - We run PCA with 50 components (default) using scanpy.
  - sc.tl.pca(adata)
  - The latent space is the space of 50 PCA components (comparable to our decipher *z* space).
  - latent = adata.obsm["X_pca"]
- **TSNE**
  - We run TSNE on the 50-dimensional PCA space using scanpy using a knn-graph with $k=10$.
  - sc.pp.neighbors(adata, n_neighbors=10); sc.tl.tsne(adata)
  - The latent space is the 2d TSNE space (comparable to our decipher *v* space).
  - latent = adata.obsm["X_tsne"]
- **UMAP**
  - We run UMAP on the 50-dimensional PCA space using scanpy using a knn-graph with $k=10$.
  - sc.pp.neighbors(adata, n_neighbors=10); sc.tl.umap(adata)
  - The latent space is the 2d UMAP space (comparable to our decipher *v* space).
  - latent = adata.obsm["X_umap"]
- **Force Atlas**
  - We run Force Atlas in scanpy using a knn-graph with $k=10$.
  - sc.pp.neighbors(adata, n_neighbors=10); sc.tl.draw_graph(adata)
  - The latent space is the 2d force-directed layout space (comparable to our decipher *v* space).
  - latent = adata.obsm["X_draw_graph_fa"]
- **scVI with batch correction**
  - We run scVI with two layers, a latent space of dimension 10, and batch correction on the *origin* label (AML vs Healthy), using scvi-tools.
  - scvi.data.setup_anndata(adata, batch_key="origin"); vae = scvi.model.SCVI(adata, n_layers=2, n_latent=10); vae.train()
  - The latent space is the 10-dimensional latent space (comparable to our decipher *z* space).
  - latent = vae.get_latent_representation()
- **scVI without batch correction**
  - We run scVI with two layers, a latent space of dimension 10, and without batch correction, using scvi-tools.
  - scvi.data.setup_anndata(adata); vae = scvi.model.SCVI(adata, n_layers=2, n_latent=10); vae.train()
  - The latent space is the 10-dimensional latent space (comparable to our decipher *z* space).
  - latent = vae.get_latent_representation()
- **Phate**
  - We run Phate using the phate Python package.
  - phate_ = phate.PHATE()
  - The latent space is the 2-dimensional latent space (comparable to our decipher *v* space).
  - latent = phate_.fit_transform(adata)
- **Harmony**
  - We run Harmony using scanpy on the PCA with 50 components.
  - sc.tl.pca(adata); sce.pp.harmony_integrate(adata, ’origin’)
  - The latent space is the 50-dimensional PCA-corrected latent space (comparable to our decipher *z* space).
  - latent = adata.obs["X_pca_harmony]
- **Seurat**
  - We run Seurat using Seurat R package.
  - adata <- FindVariableFeatures(data); adata.list <- SplitObject(adata, split.by ="origin"); features <- SelectIntegrationFeatures(object.list = adata.list); adata.anchors <- FindIntegrationAnchors(object.list = adata.list,, anchor.features = features);adata.combined <- IntegrateData(anchorset = adata.anchors); adata.combined <- ScaleData(adata.combined, verbose = FALSE)
  - The latent space is the PCA-corrected latent space (comparable to our decipher *z* space).

#### Application of Decipher to PDAC data

We applied Decipher to data collected by Burdziak et al. [52], consisting of PDAC samples from mouse models with and without KRAS mutation. We subsetted the data to cells undergoing acinar-to-ductal metaplasia (ADM), from three conditions: normal stress, normal, and KRAS-mutated. For our results in Fig. 3, we used 10 latent dimensions (*z*), 2 Decipher components (*v*), and $\beta = 0.1$. All other parameters were default, and the model was run with early stopping. We rotated the resulting Decipher embedding such that the Decipher 1 axis aligned with acinar-to-ductal maturation. Since the desired path of trajectories was previously known, we manually defined trajectories the normal and KRAS-mutated conditions by specifying the order of the clusters.

To interpret the latent dimensions, we selected the latent *z* component that yielded the greatest significant separation between KRAS-mutated and non-mutated cells. Degree of separation was quantified by T-testing the distribution of a factor over the KRAS-mutated population and the non-mutated population; the absolute value of the T-statistic was used for selecting the best separating component. We also computed the correlation across cells between each latent dimension and each gene (Additional file 3: Table 1). This resulted in a list of genes ranked by correlation values for each latent dimension that may be analyzed either individually, or using gene set enrichment analysis (GSEA) (Fig. 3e, Additional file 3: Table 2). For individual gene analysis, we looked at genes from the Kras-mutated signature from Burdziak et al. [52], as well as p53 targets from Fischer [57]. To demonstrate that a small set of genes (such as the Kras targets) are ranked significantly higher compared to the ranking distribution of all genes, we applied a Wilcoxon rank-sum test between the set of genes and all genes. Finally, we show the relationship between latent components (*z*) and Decipher components (*v*) through visualization the correlation between each *v* and *z* (Fig. 3c).

We repeat the above process using scVI as a comparison. ScVI was run with the same data, using 2 layers and 10 latent layers, with the gene likelihood parameter set to “nb.” The two-dimensional visualization of scVI was obtained using UMAP visualization (Fig. S1d). We repeat the same analyses as above on the scVI latent components, identifying the best separating latent component and running GSEA (Fig. S1e) to compare the interpretability of the two methods. We demonstrate that the T-statistics quantifying the degree of separation between KRAS-mutated and non-mutated cells in Decipher’s latent components is higher than in scVI’s latent components, by plotting the sorted T-statistics for each significant component (Fig. 3f).

#### Application of Decipher to AML patient data

Before applying Decipher to the AML patient data, we first performed a gene filtering step to include the most important genes representative of all cell types. For each patient, we performed PhenoGraph [5] clustering using 40 principal components and 30 neighbors. Then, using scanpy’s [103] *rank_genes_groups()* function, we performed a T-test to identify the top 400 most differentially expressed genes for each cluster. This list of genes was pooled with a list of known marker genes to produce the final set of genes on which the model was run. Including known marker genes is optional; we did so to facilitate their visualization in downstream analysis. Similarly, the gene-filtering steps are optional, and we showed that Decipher would still identify valid trajectories without any gene filtering (Supplementary Information; Fig. SI1).

We also removed erythrocytes and lymphocytes from the data, as they were not relevant for the analysis of AML derailment.

We obtain the following.

- 3130 genes for the joint dataset AML1 and normal
- 3264 genes for the joint dataset AML2 and normal
- 3258 genes for the joint dataset AML3 and normal
- 2863 genes for the joint dataset AML13 and normal
- 2532 genes for the joint dataset AML14 and normal
- 3291 genes for the joint dataset AML15 and normal
- 2944 genes for the joint dataset AML16 and normal
- 2664 genes for the joint dataset AML17 and normal

Aside from these filtering steps, we emphasize that no other pre-processing or normalization was performed, as the model is always run on raw counts data. For our results (Fig. 4), we use latent factors *z* of dimension 10, Decipher components *v* of dimension 2, a neural network decoder from *v* to *z* with one hidden layer of dimension 64, a linear decoder from *z* to *x*, a neural network decoder from *x* to *z* with one hidden layer of dimension 128, and another neural network decoder from (*z*, *x*) to *v* with one hidden layer of dimension 128. BatchNorm was applied after each hidden layer in the neural networks, followed by a ReLU activation. We set $\beta = 0.1$ and a batch size of 64. The code to reproduce the results in this manuscript is available at https://github.com/azizilab/decipher_reproducibility.

#### Interpretation of Decipher components, latent dimensions, and basis

To identify pathways associated with the latent components of Decipher, we computed the covariance of each gene with each of the Decipher components, the latent dimensions, and the results from basis decomposition (Additional file 2: Table S2). Precisely, we computed for each gene *g* the covariance over cells between $x_g$ and each variable $v_1, v_2$ and $z_j$ for $j \in [10]$. We then ran gene set enrichment analysis (GSEA) [130], with genes preranked by covariance with each latent component. Next, to interpret the learned basis functions, we ranked genes by their weights in each basis to identify pathways most associated with each basis. For all use cases, GSEA was run on the pre-ranked setting against the Hallmarks Database, with 1000 permutations and no collapse (Additional file 3: Table 4). To select genes for visualization in Fig. S6a, we selected a top pathway for each component/basis function with known biological importance and found the top disrupted genes belonging to that pathway.

We highlight the usage of Decipher in reconstructing gene patterns over a temporal dimension. These analyses necessitated the translation of cell-level metadata to the temporal dimension. In order to analyze observations such as cell type and mutation proportions, along the temporal dimension, we applied the projection method outlined in the trajectory inference section to obtain a cell-level Decipher time. This transformation allowed for observations to be directly studied along the temporal axis. For discrete observations such as cell type, we first performed nearest-neighbor smoothing using Scikit-learn 0.24.0 [131], with 50 neighbors and a radius of 0.2. A smoothed label was obtained for each cell by taking the mode of the labels among its neighbors. We then visualized the observations along a temporal axis by producing a scatterplot of cell observations, where the x-axis is the computed pseudotime of each cell and the color corresponds to the smoothed label (Figs. 6b, c, S9).

A key feature of Decipher is its ability to produce Decipher components that can be rotated to align with axes of disease maturation and development. We extended our analyses of the NPM1 mutational status of cells to examine its correlation with Decipher component 2 in AML1, 2, and 3. Specifically, we specified a cutoff threshold of 0.4 for the mutation proportion and classified cells with proportions greater than that as belonging to the mutated class and cells with proportions less than as being wild-type. We then binned cells by their pseudotime projections (with a bin size of 0.5 and a sliding window of 0.05). We then counted the number of cells classified into mutated and wild-type by our threshold in each bin and smoothed the resulting counts by time curves using a 1d Gaussian kernel (using scipy [129]) with a standard deviation of 2. We visualized the results as distributions along the Decipher component 2 axis to emphasize the shift in NPM1 mutational status (Fig. 4f, Fig. S3a).

#### Application of Decipher to TET2 and DNM cohorts

To evaluate the performance of Decipher at the cohort level in AML (Fig. S3c), we first ran the model on the concatenated data from AML1, AML2, and AML3 (without the added healthy reference), with lymphoids, erythroids, and MEP cells removed. We ran the model with standard settings, and $\beta$ = 0.001. We then defined cell clusters using 15 neighbors and leiden resolution = 0.7, and manually specified the three trajectories corresponding to leukemic maturation in each patient by listing the order of clusters. This then allowed for the plotting of specific gene markers of interest. The same process was repeated for the combination of the TET2 and DNM cohorts (Fig. S3d), but with $\beta$ = 0.01.

#### Comparison of disrupted genes across patients

To determine if disrupted mechanisms between healthy and AML disruption were shared across patients, we first obtained combined disruption scores for each patient as detailed above. We limited our analysis to transcription factors and identified the top disrupted transcription factors in each patient in order to identify top shared disrupted gene programs. We also visualized disruption scores between patients as a 3D scatterplot of individual patients (Fig. 6d), or by taking the mean of patients with similar mutational statuses (Fig. S10e).

#### Distribution of TF peak expression over time

To study the patterns of TF expression over time, we directly utilized the gene patterns produced by Decipher that showed the expression values of each gene over the learned Decipher time axis. For each TF present in the data, expression patterns were first smoothed using a 1d Gaussian filter (using scipy [129]) with the standard deviation of the Gaussian kernel set to 3. This smoothing is performed only for detecting peak expression and is not applied to expression plots. Local maxima of expression were then identified by searching for points at which the first derivative of the curve switches from positive to negative. We furthermore filter points by using the midrange of expression (defined as the mean of the minimum and the maximum expression) as a threshold: local maxima whose expression values were less than this value. We additionally included the starting point as a maxima if the expression value was greater than the threshold and the first derivative was negative or if the maximum value of the expression was at the start; we included the ending point as a maxima if the maximum value of expression was at the end. For visualization, we plotted the kernel density estimation (with a bin width of 0.05) of all TF maxima along the Decipher time axis to show the points in time where overall TF activity is concentrated (Fig. S6). Kernel density estimation was performed using seaborn [132], and plotting was done using matplotlib [133].

#### Analysis of temporal TF co-regulation

In conjunction with the visualization of the timing of TF activity, we also sought to determine if families of similar TFs demonstrated coordinated activation times and if these temporal dynamics could be utilized to derive insight into regulatory wiring. We focused our analyses on the top 20 most disrupted TFs by the combined disruption metric, as well as the known disrupted TFs from the literature. The Decipher gene pattern for each TF was visualized as rows in a heatmap, with the horizontal axis representing the pseudotime axis and the color representing the z-scored expression value. The rows were sorted based on the time at which the maximum peak occurred, and the TFs were labeled with colors based on their biological function (Fig. 6b, c; Fig. S9a, b).

#### Estimation of uncertainty in expression patterns

Because Decipher is a probabilistic model, it learns the uncertainty about the gene expression induced by a cell representation *v*. Given a location *v* in the Decipher space, the distribution of the expected gene expression $\mu _{g}(v)$ of gene *g* in a cell with representation *v* is given by,

$$\begin{aligned} \mu _{g} | v ~\sim ~ h(z)_g | v \end{aligned}$$

where $$z|v \sim \mathcal {N}(f_{\text {mean}}(v), f_{\text {var}}(v))$$.

To compute this uncertainty for each *v*, we sample 100 values for *z* from $z | v \sim \mathcal {N}(f_{\text {mean}}(v), f_{\text {var}}(v))$ and compute $\mu _{g} = h(z)_g$ for each of them. In Fig. 5b, the shaded bands represent the interquartile range (25–75) of the 100 samples.

#### Analysis of bulk AML data

We applied DeSeq2 [134] to obtain metrics characterizing the AML data at the cohort level. Using the resulting L2FC and *p* values, we were able to confirm whether or not expected genes, as well as our newly identified disrupted genes, were also differentially detected in the bulk data. For both sets of genes, we selected genes whose absolute L2FC was greater than 1 and reported the maximum *p* value.

#### Application to gastric cancer evolution

We applied Decipher on the gastric cancer data from Kim et al. [84]. We pooled the data from the 24 patients in the study, each with pre-malignant cells and cancerous cells. Nine of these patients have *intestinal* cancer and the other 15 patients have *diffuse-like* cancer. The resulting data has 12,612 cells and 8705 genes. We ran Decipher with its default hyperparameters: *z* of dimension 10, *v* of dimension 2, and for 30 epochs (Fig. 7b–d).

In sum, Decipher on the gastric data was applied on

- 12,614 cells
- 8705 genes
- From 24 patients
- With 2 major types of cells (pre-malignant, cancerous)
- With 2 types of cancer (diffuse-like or intestinal)
- With different stages of cancer

## Supplementary information

Additional file 1. Supplementary figures S1 to S15 and supplementary information.

Additional file 2. Supplementary tables S1 and S2.

Additional file 3. Tables 1 to 4.

## Data Availability

The data discussed in this manuscript is deposited in the National Center for Biotechnology Information’s Gene Expression Omnibus (GEO), with accession ID GSE298955 for the RNA-seq [135] and accession ID GSE299002 for the ATAC-seq [136]. The bulk RNA-seq data for AML patients is publicly accessible at GEO with accession IDs GSE49642, GSE52656, GSE62190, GSE66917, GSE67039, and GSE106272 [137]. Bulk RNA-seq for normal HSC-enriched subpopulations is accessible at GEO ID GSE48846 [138]. Decipher is available at https://github.com/azizilab/decipher [139]. The specific code that produced the results and figures of this manuscript is available at https://github.com/azizilab/decipher reproducibility [140]. The Decipher model has also been implemented as part of scvi-tools: https://scvi-tools.org [104]. The dataset of mouse pancreatic cancer cells is available from [53]. The dataset of human gastric cancer cells is available from [141].

## Footnotes

1 Because the Gaussian process is used here only to define the distribution of the observations, and not to sample an unobserved latent variable, there is no computational difficulty in using it.
