# Supplementary material for: Joint representation and visualization of derailed cell states with Decipher
Source: Genome Biol. 2025 Jul 23;26:219. doi: 10.1186/s13059-025-03682-8 (PMC12285193; doi:10.1186/s13059-025-03682-8)
Supplement: Supplementary file 1 — Additional file 1. Supplementary figures S1 to S15 and supplementary information. [file 13059_2025_3682_MOESM1_ESM.docx]

Joint representation and visualization of derailed cell states with Decipher.

Supplementary figures S1 to S15 and supplementary information.


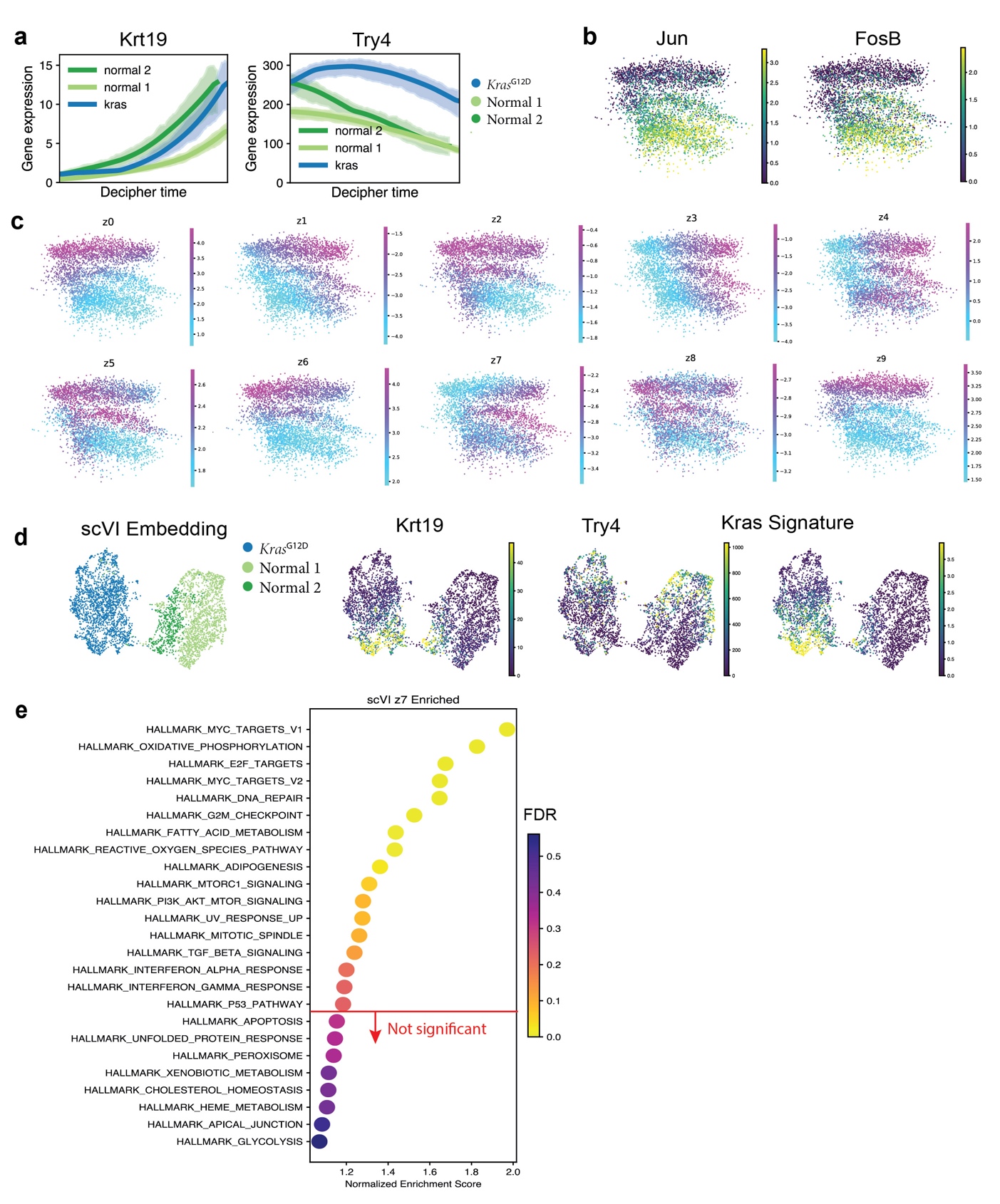


**Fig. S1. Comparison of latent spaces of scVI and decipher. (a)** Expression of ductal marker *Krt19* and acinar marker *Try4* over learned Decipher time. **(b)** Decipher embedding colored by AP-1 factor genes *JUN* or *FOSB*. **(c)** Decipher embedding colored by latent factors. **(d)** 2D UMAP embedding computed from scVI latent factors, colored by mutational status, gene markers, and the *Kras*-mutated signature. **(e)** Pathways enriched by GSEA for z9 in scVI, which is the factor with the biggest separation of *Kras*-mutant and normal cells, show *Kras* signaling both up- and downregulated.


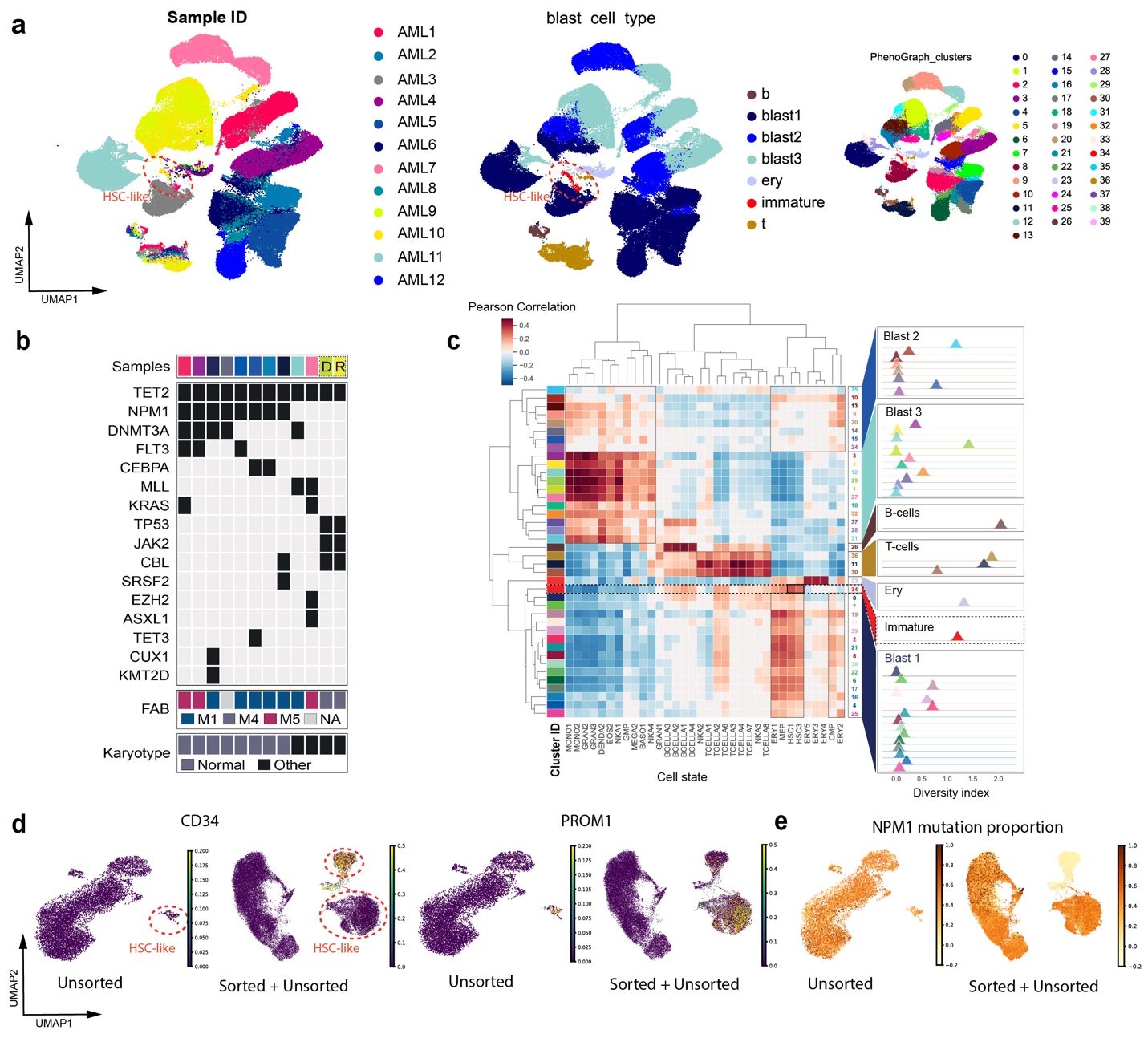


**Fig. S2. Characterization of AML samples and immature cell enrichment. (a)** UMAP projection of unsorted bone marrow transcriptomes from 11 AML patients (AML9 and AML10 are from the same patient). Cell colored by patient ID, cluster ID, or annotated cell type. **(b)** Targeted mutation, karyotype, and morphological (FAB) assessments of patient bone marrow samples (**Table 3**). D and R, respectively, denote pairs of diagnosis and relapse samples from the same patient. **(c)** Left, Pearson correlations between cluster centroid expression and bulk gene expression data from sorted subsets of healthy HSPCs[[64]](https://paperpile.com/c/jP4e1f/oP7o3) (Methods). Right, the Shannon Diversity index computed for the distribution of patients in each cluster, controlling for cluster size (Methods). Higher diversity indicates a greater mixing of patients. **(d)** UMAP projection of AML1 single-cell transcriptomes including or not including sorted CD34^+^/PROM1^+^ cells. Cells are colored by the expression of CD34 and PROM1 and **(e)** by the proportion of *NPM1* mutation in their neighborhood (Methods).


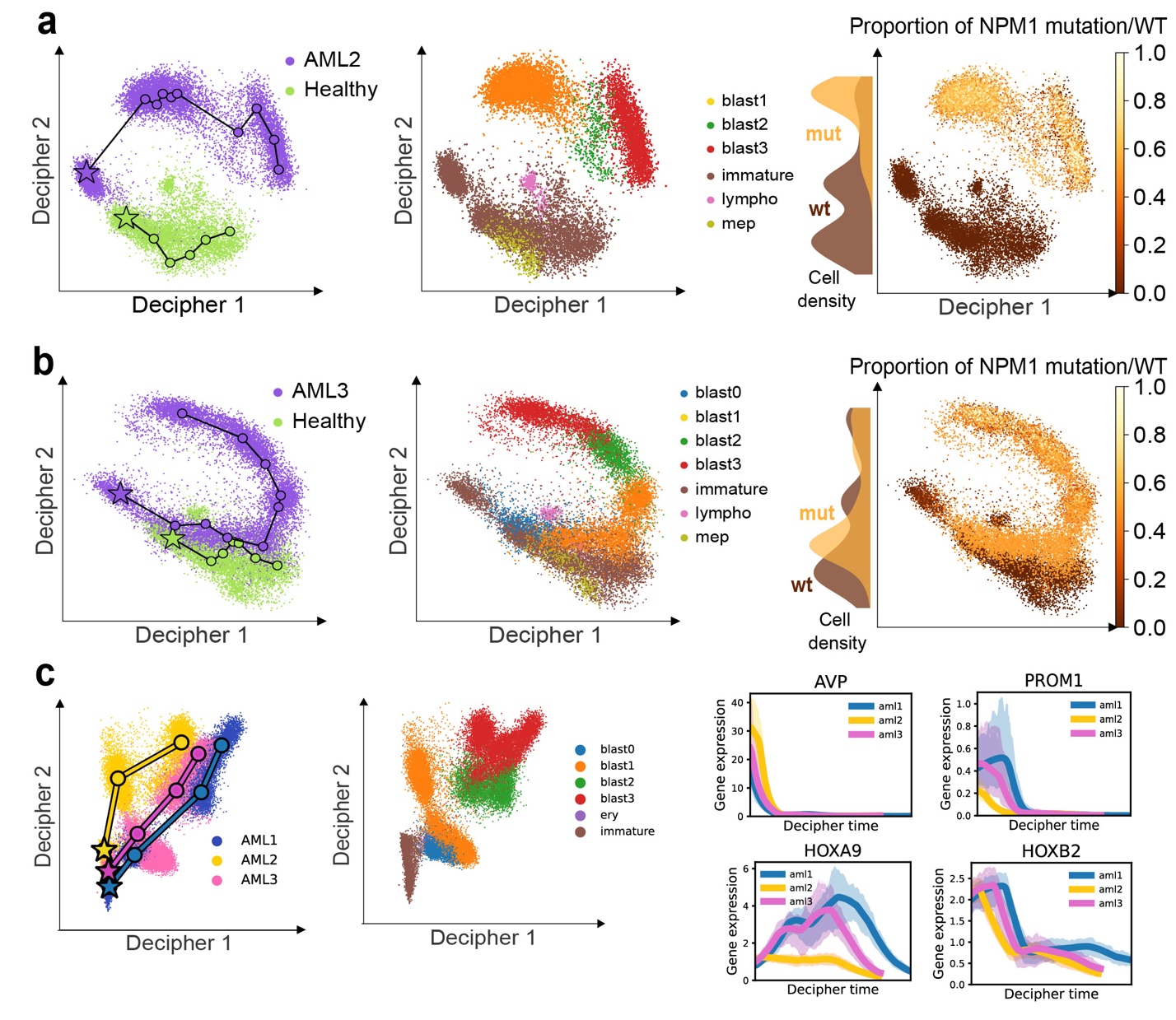


**Fig. S3. Application of Decipher to other AML patients. (a,b)** Decipher space visualization of cells from the healthy donor and AML2 **(a)** or AML3 **(b)**. Cells colored by condition (left), cell type (middle), and proportion of *NPM1* mutation (defined as the number of neighbors with mutation/all neighbors for each cell, using 30 neighbors) (right). Lines define trajectories for normal and healthy samples (left). **(c)** Decipher space visualization of AML cells from AML1, AML2, and AML3 combined colored by sample origin and cell type (left), with expression of key gene markers along the maturation trajectory denoted by the marked trajectories (right).

**Figure S4. Integration of AML and normal samples with existing methods. (a,b)** Projection of 37,395 cells from patient AML1 and from a healthy donor using different tools (Methods). Each dot represents a cell colored by origin (left) and type (right). Non-batch-correcting visualization methods tSNE, UMAP, and ForceAtlas.  (a), and batch-correcting methods – UMAP of the Seurat space, scVI space, and Harmony space (b) – they all fail to integrate AML and healthy cells. Lines indicate explicit trajectories inferred on the scVI space (Methods).


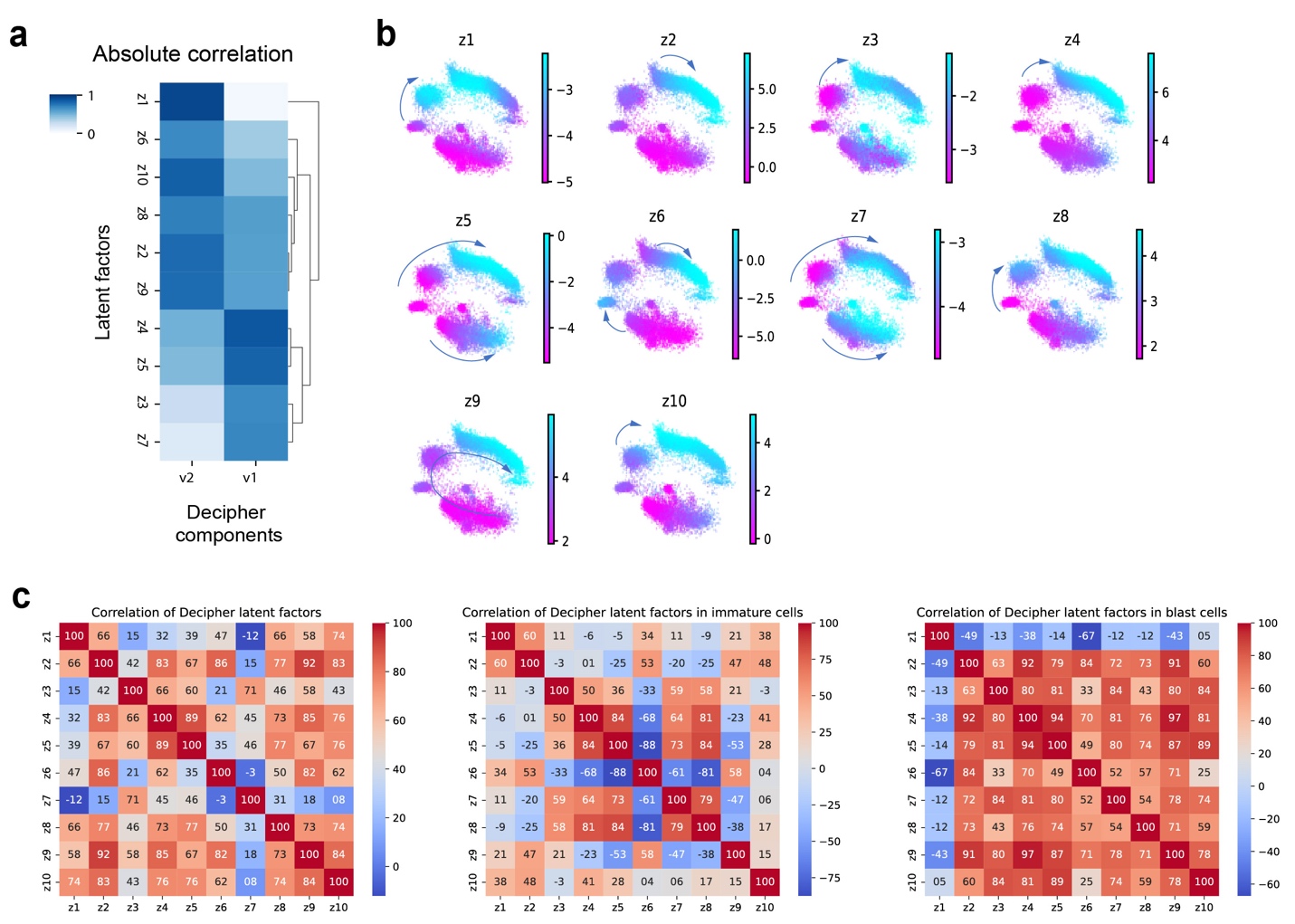


**Fig. S5. Latent factors capture AML cell-state transitions. (a)** Absolute value of correlation between Decipher components and latent factors. **(b)** Decipher space colored by all latent factors for AML1 (a subset of these are shown in **Fig. 4h**). Arrows indicate cell-state transitions corresponding to an increase in the latent factor. **(c)** Correlation matrices between the Decipher latent factors computed from all cells, only immature cells, or only blast cells. The correlation structure changes between cell states. The correlations are multiplied by 100 to be on a scale -100 to 100.


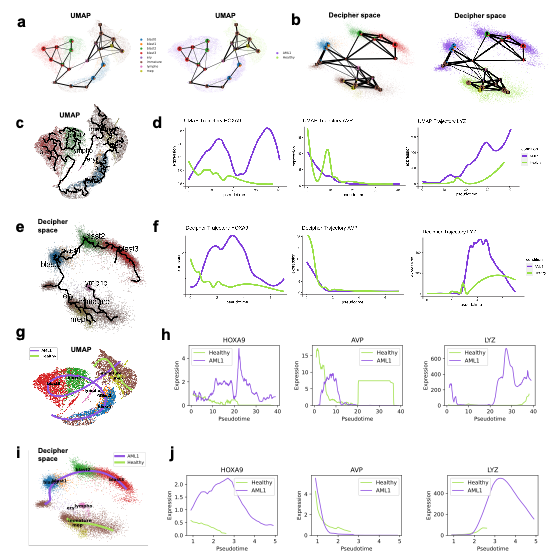


**Fig. S6. Trajectory inference methods on the Decipher space and their default space. (a,b)** Explicit trajectories inferred using PAGA with its default dimensionality reduction method (a) and with the Decipher space as input (b). The trajectories fail to link blast 1 to blast 2 cells and incorrectly connect healthy immature cells to late-stage blast 3 cells. **(c-j)** Trajectories from Monocle (c,e) and Slingshot (g,i) from their default dimensionality reduction method (c,g) and from the Decipher space (e,i); and their associated gene patterns (g,f,h,j). The default trajectories (c,g) are incorrect and distort the ordering of cells, e.g., they all connect immature and blast 3 cells. The Decipher-based trajectories are consistent with the biologically validated trajectories (**Fig 4c**).


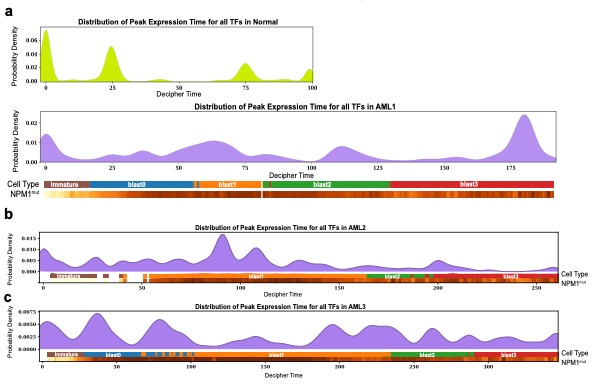


**Fig. S7. Temporal distribution of genes and TFs. (a)** Timing of peak expression of TFs on the Decipher time axis in normal (top) and AML1 (bottom) samples. Density plots display the local maxima across all TFs in each sample. Distribution of local density maxima across all TFs along Decipher time in AML2 **(b)** and AML3 **(c)**.


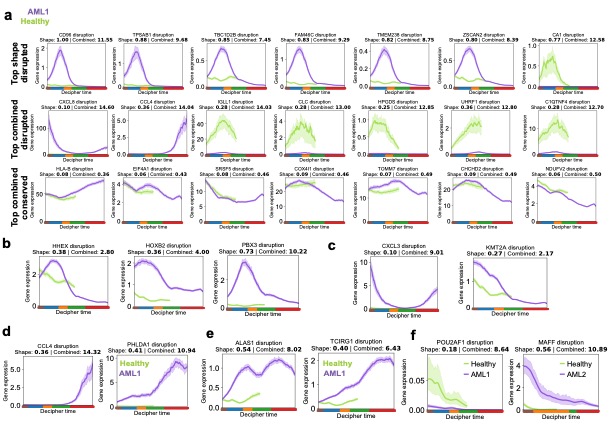


**Fig. S8**. **Gene trend analysis using Decipher. (a)** Reconstructed expression patterns for top disrupted/conserved genes that were identified using the shape and combined disruption metrics. **(b–e)** Reconstructed expression trends and disruption scores for homeobox genes and cofactors (b), genes upregulated in immature leukemic cells (c), TNFɑ pathway (d), oxidative phosphorylation (e) in AML1. **(f)** Reconstructed trends and disruption scores for genes disrupted mainly in an early immature state and thus unidentified in bulk analysis.


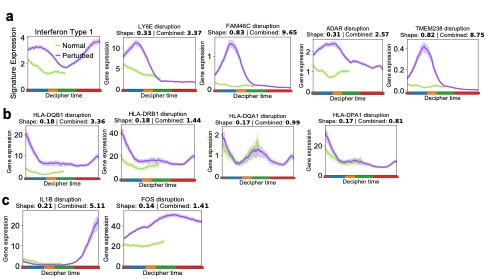


**Fig. S9**. **Notable genes co-expressed with aberrant TFs and comparison of TFs across patients. (a)** Reconstructed expression trends for IFN type 1 signature (left) and individual genes (right) found to be co-expressed with *HOX* TFs under both normal (green) and AML (purple) conditions. **(b,c)** Reconstructed expression of genes involved in IFN type 2 (b) and TNFɑ pathway (c).

**Fig. S10**. **Altered TF dynamics in patients AML2 and AML3. (a,b)** Timing of TF expression along Decipher time in AML2 (a) and AML3 (b) samples. Heatmaps show log-transformed and z-scored expressions for the top 20 TFs with the highest combined disruption scores in each sample and known TFs from literature, sorted by timing of peak expression. Colorbars correspond to cell type and proportion of *NPM1*-mutated cells among the 30 nearest neighbors of each cell; both are smoothed over the 50 nearest neighbors in the Decipher space.


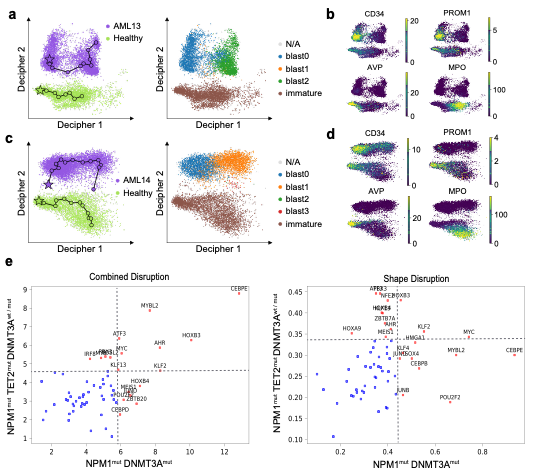


**Fig. S11. Decipher characterizes a *DNMT3A*-mutated AML cohort.** **(a–d)** Decipher space embedding of sorted cells from two *DNMT3A*^mut^ AML patients (Table 3), colored by the sample of origin and cell state (a,c) and by marker gene expression (b,d). AML maturation is observed as a decrease in *CD34* expression and normal HSPC differentiation as a decrease in *AVP* and an increase in *MPO*. Decipher identifies and aligns myeloid trajectories in AML even when cell states are missing (blast1,3 in AML13; blast2,3 in AML14). **(e)** Comparison of disrupted genes in patients bearing *NPM1* and *DNMT1* mutations, with and without *TET2* mutation, based on combined disruption and shape disruption scores. Red points represent TFs in the 80th percentile of disruption in at least one category. The dotted lines show the 80th percentile threshold.


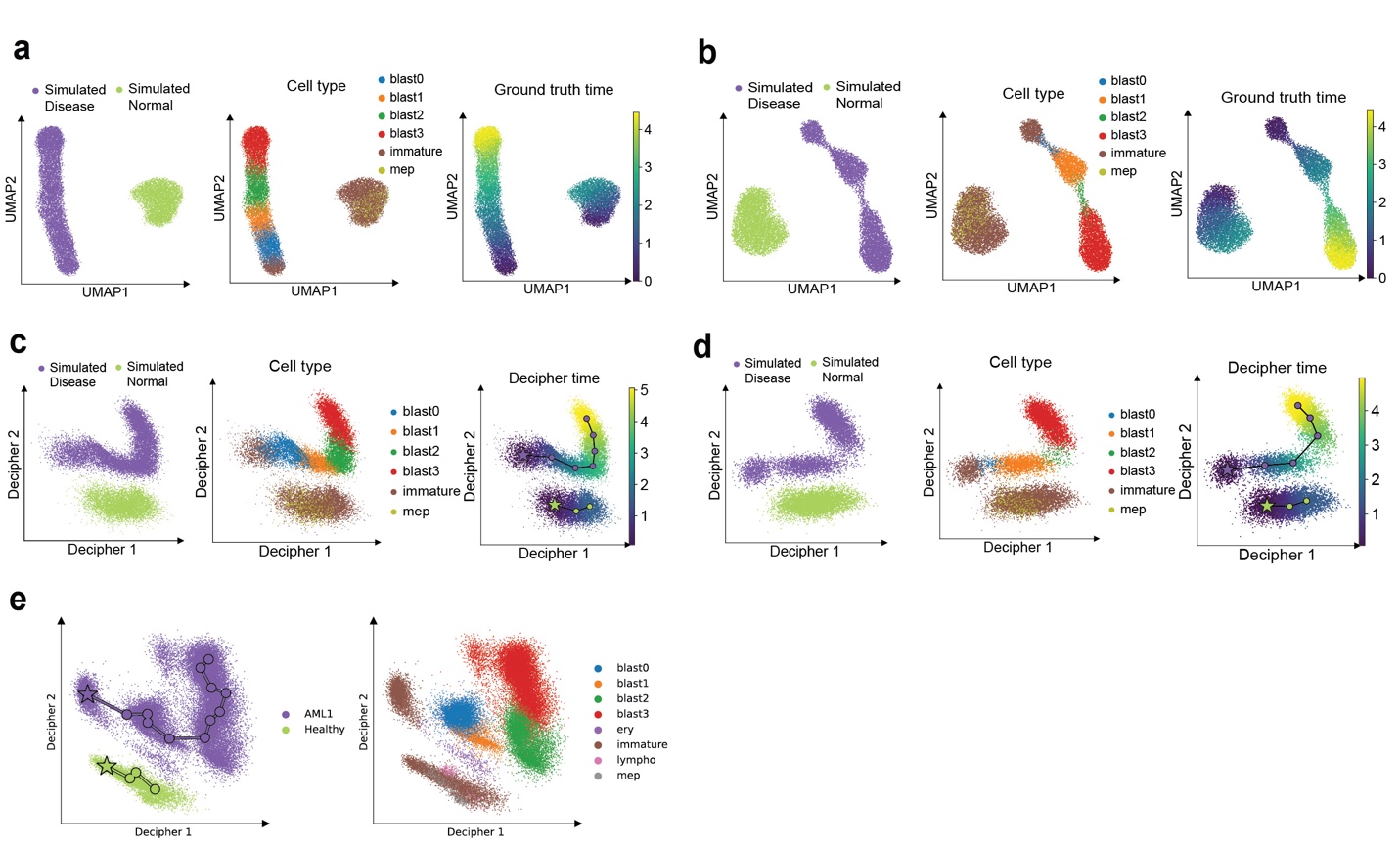


**Fig. S12. (a)** UMAP visualization of semi-synthetic data (18,000 cells) simulating an AML patient and a healthy reference (Methods) along simulated trajectories. Each dot represents a cell colored by sample (left), cell type (middle), and ground truth time (right). **(b)** UMAP visualization of the data simulating rare cell types. Similar to (a) but removing 90% of blast0 (blue) and blast2 (green). Each dot represents a cell colored by sample (left), cell type (middle), and ground truth time (right). **(c)** An example of Decipher applied to the data in (a). It preserves the order of cell states and shows the shared maturation (Decipher1) as well as the divergence of mature leukemic blast cells (blast2,3) in AML (Decipher2). Each dot represents a cell colored by sample (left), cell type (middle), and inferred Decipher time (right). **(d)** Decipher applied to data shown in (b) can still learn meaningful representation and trajectories. **(e)** Decipher space with derailment trajectories on the AML1 and healthy patient data, trained on the full 10,988 genes without gene filtering. The trajectories are equivalent to those found by Decipher in the filtered data (**Fig 4c**).


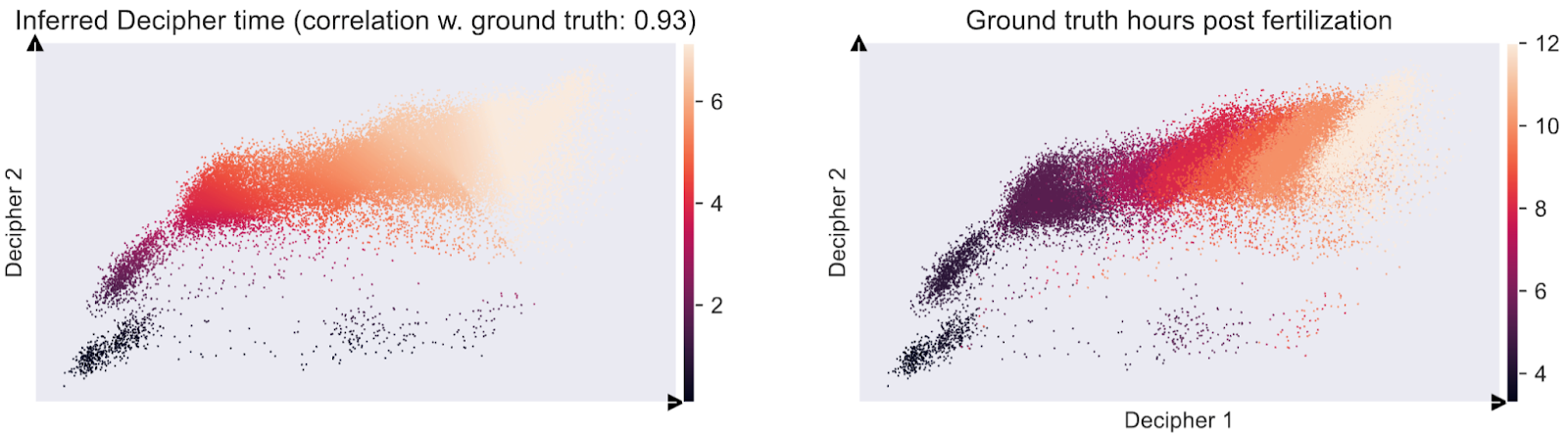


**Fig. S13**. **Decipher space learned for the 38,731 cells from the zebrafish embryogenesis data.** Each point represents a cell, colored by its inferred Decipher time (left) or collection time point (right). The Decipher space organizes cells precisely along the expected progression of zebrafish embryogenesis, and the inferred Decipher time demonstrates a strong correlation with the true ground truth developmental time.


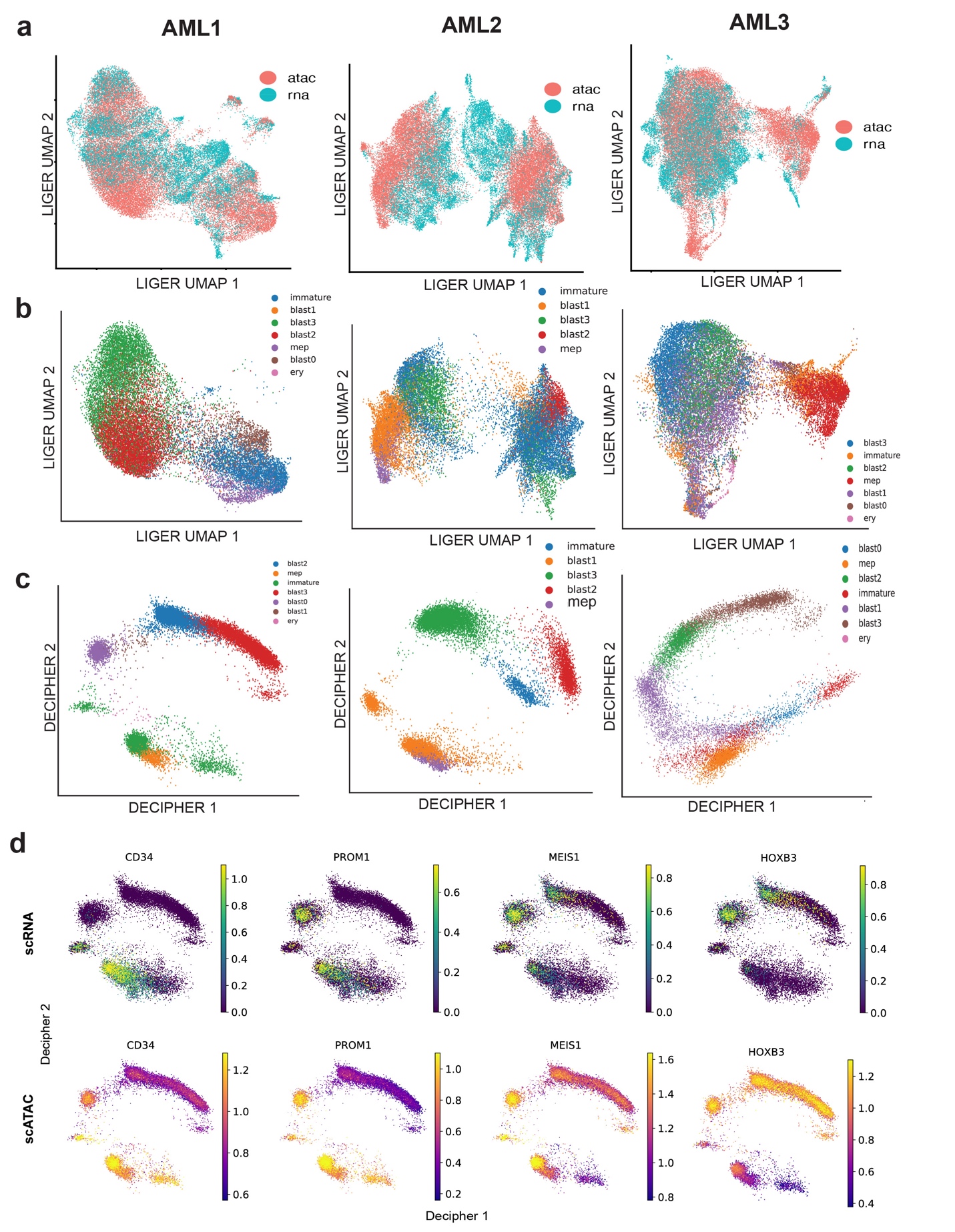


**Fig. S14**. **Integration of scRNA and scATAC data (a)** UMAP embedding of cells from scATAC and scRNA-seq in the LIGER integrated space in three AML patients. **(b)** UMAP projection of cells colored by cell types in ATAC-seq in the LIGER Integrated space in three AML patients. **(c)** Projection of cells in scATAC data in 2D decipher space colored by cell type. **(d)** scRNA and scATAC-seq of key marker genes in AML1. Scatterplots show expression (top) and accessibility (bottom) in the Decipher space (Methods).

**Fig. S15**. **TFs with enriched motifs in all patients.** scRNA and scATAC-seq of TFs found to have enriched motifs in the vicinity of late TFs (expressed after NPM1 mutation) in two out of three AML patients (Methods). Scatterplots show expression (top) and accessibility (bottom) in the Decipher space for AML1 **(a)**, AML2 **(b)**, and AML3 **(c)**.

**SUPPLEMENTARY INFORMATION**

**Semi-synthetic simulation and robustness to gene-filtering**

To further complement our validation of Decipher’s performances, we created semi-synthetic data that is more realistic than purely simulated data by leveraging the AML data. We used the Decipher model trained on real data (normal HSPCs and sorted cells from AML1) to obtain realistic values for the probabilistic model. Then we simulated new data by (i) forming two divergent ground-truth trajectories in the Decipher component space (Methods), (ii) generating synthetic cells by sampling their Decipher components uniformly along these trajectories, and (iii) generating synthetic gene expression values for these simulated cells using our probabilistic model (**Fig. 1d; Fig. S12a,b**). Then, we fitted a new Decipher instance to this simulated dataset that is realistic and for which we have ground truth. Decipher successfully learned a latent space in which cell-state transitions from immature to blast0–3, and both normal and AML trajectories are correctly aligned along the Decipher 1 component (**Fig. S12c**). The derailment in the AML condition is captured by the Decipher 2 component, which precisely locates the bifurcation point. We assessed the recovery of cell order by evaluating the correlation between the inferred Decipher time and the ground-truth trajectory. To confirm the robustness of these results, we repeated the entire Decipher pipeline across five simulated datasets and consistently observed a correlation above 0.94. Finally, we examined robustness in the context of rare or poorly represented cell states, similar to the fully synthetic experiments. We removed 90% of cells from two intermediary cell states (**Fig. S12c**) and fitted Decipher on this new simulated data. Even with a non-uniform sampling of cell states, Decipher successfully preserved the order of cell states (**Fig. S12d**), as found in the fully synthetic experiments.

In the main analysis, we filtered genes down to about 3,000 as a standard pre-processing step of single-cell data (see Methods). To understand the sensitivity of Decipher to the gene-filtering step, we have reproduced the main analysis (**Fig 4i**) with the entirety of all genes for the healthy and AML1 patients. Since Decipher is designed with performance in mind, it is possible to train Decipher on all the 10,988 genes in less than 5 minutes. We obtain similar Decipher spaces and equivalent trajectories, thereby illustrating the robustness of Decipher to the absence of the presence of gene pre-filtering (**Fig S12e**).

**Evaluation of Decipher on a real-world developmental dataset collected at different time-points**

We evaluated Decipher on the task of comparing healthy and perturbed samples, and showed its superiority over other methods to correctly capture derailment between the two conditions. Now, we measure how well Decipher recovers developmental trajectory from data collected at different time-points.

We use publicly available data for zebrafish embryogenesis (Farrell et al., 2018), which profiles 38,731 cells collected from 12 closely spaced developmental time points spanning 3.3–12 hours post-fertilization (hpf). We select 13,296 of 17,239 genes by filtering for expression in at least 100 cells, but keep all cells and do not apply any additional pre-processing. Decipher clearly organizes the cells along a visual developmental trajectory. The Decipher time along that trajectory agrees very closely with ground truth time point labels (Spearman correlation = 0.93) (**Fig. S13**).

**Design of AML Experiment**

To characterize the impact of NPM1mut while minimizing confounding effects, we focused on patients bearing TET2 epigenetic mutations (n = 12), with and without NPM1 mutations. We used scRNA-seq to profile 104,116 AML bone marrow cells (**Fig. S2a** and **Table 3**). Assuming that leukemic cells preserve a subset of normal hematopoietic differentiation programs, we used well-characterized normal references [[64]](https://paperpile.com/c/jP4e1f/oP7o3) to achieve cluster-level annotation of the maturation stage (**Fig. S2b**; Methods). This analysis revealed a broad spectrum of leukemic cell states that partially recapitulates the diverse morphological subtypes in our cohort [[103,104]](https://paperpile.com/c/jP4e1f/3qDCQ+XoTct) (**Fig. S2c**); differentiated (M1) samples were mostly populated by immature blasts, myelomonocytic (M4) and monoblastic (M5) samples by cells with greater maturation. However, most samples contained many cell types.

Comparison of pre-leukemic cell and normal HSC phenotypes consistently returned PROM1 as a marker for the immature population in human NPM1mut AML samples. This analysis also identified consistent underexpression of HSC genes such as CD34 and AVP in NPM1mut cells (**Fig. S2d,e**), concordant with the demonstrated ability for NPM1 mutations to induce HOX genes in vitro [[105]](https://paperpile.com/c/jP4e1f/QLQHj) and confer self-renewal properties to myeloid progenitors in mice [[59]](https://paperpile.com/c/jP4e1f/aKoWn). Overall, this approach provides an expanded map of both frequent and rare cellular populations in each patient and unambiguously delineates NPM1 mutation status.

**Analysis of single-cell ATAC-seq**

To further study altered TF activity in AML, we performed an integrated analysis of gene expression and chromatin accessibility patterns via single-cell Assay for Transposase-Accessible Chromatin (scATAC-seq; Methods). Using scATAC data, we explored possible regulatory relationships between disrupted TFs expressed in early immature cells and those expressed later in AML progression that could explain the observed cascades of TF activity (Fig. 6d; Fig. S14). Specifically, we identified each patient's top 50 disrupted TFs and split them into those expressed before and after NPM1 mutation, labeled as ‘early TFs’ and ‘late TFs’, respectively. We then investigated putative regulatory relationships between the two groups of TFs by computing a motif enrichment score for each early TF detected within accessible regions in the vicinity of late TFs as targets. Then, using bootstrapping analysis, we compared the motif scores to a null distribution generated from same-size random sets of target TFs that are expressed in at least 1% of cells (Methods). In all three patients, we found significant motif enrichment in HOX TFs, including HOXB4 (p < 0.0458), HOXB6 (p < 0.032), and FOSL1 (p < 0.0057), suggesting their putative role in regulating TFs driving AML transformation. Two out of three patients have significant motif enrichments in GATA2, MYC, NR4A2, MAFF, ATF3, NFKB2, RELB, KLF2, and ZNF274 (p < 0.05; Methods).

We then examined the dynamic accessibility of the TFs themselves during AML progression by integrating scRNA and scATAC-seq data using LIGER[[36]](https://paperpile.com/c/jP4e1f/B5aMB) (Fig. S15a,b, Methods). Through this process, we are able to project scATAC data into the 2D Decipher space inferred from scRNA (**Fig. S14c**), finding that the patterns of chromatin accessibility mirror the gene expression dynamics for regulators with enriched motifs (**Fig. S14d**). We find that the significantly enriched motifs (GATA2, MYC, NR4A2, MAFF, ATF3, NFKB2, RELB, KLF2, ZNF274) show altered expression in immature cells in two out of three patients (**Fig. S15**).

Interestingly, motif enrichment analysis using scATAC data further showed enrichment of motifs for known interferon-induced TFs such as STAT1 (MA0137.1,p<0.0357) and IRF1 (MA0050.1,p<0.0057; MA0050.2,p<0.0216) among the top disrupted TFs in AML1 and AML3. Our approach thus resolves the timing of TF activity concerning significant events such as genetic mutations and activated signaling pathways, guiding further studies of regulatory relationships.

**Analysis of single-cell ATAC-seq**

We utilized ArchR[[106]](https://paperpile.com/c/jP4e1f/Nzqq) for pre-processing scATAC-seq data. To analyze accessibility trajectories from immature cell states to mature blast cells, we combined each patient AML sample with scATAC-seq data collected from a healthy bone marrow sample. ArchR was run using default parameters and the HG38 genome for peak alignment. UMAP visualization was run using ArchR's implementation with iterative LSI to provide a preliminary visualization of the distribution of cells in the ATAC space. The peak counts and gene scores were exported and stored in an AnnData object for downstream use in *Scanpy*.

**Integration of scATAC and scRNA**

To integrate the scATAC data with the scRNA data, we applied LIGER[[36]](https://paperpile.com/c/jP4e1f/B5aMB), using the gene scores from ArchR and the counts from the scRNA data as the two inputs. We applied a log2(*x*+1) transformation to the gene scores (where x=gene scores) to improve the signal. Running with standard parameters yielded an integrated space that could be visualized using UMAP (**Fig. S14a,b**) to confirm the overlapping of modalities and improved separation of cell types. From this step, we exported the matrix of normalized cell factors, *H*, representing the LIGER shared factors across modalities that can be subsequently analyzed to compare cells from the scATAC data to cells in the scRNA data.

**Projection of Accessibility scores in Decipher space**

The first step of this analysis was to project cells in scATAC-seq to the Decipher space by finding the most similar cells in the RNA-seq to each cell in the ATAC-seq, to transfer metadata and Decipher values from the RNA-seq to the ATAC-seq. To achieve this, we applied a nearest neighbors algorithm using Scikit-learn [[107]](https://paperpile.com/c/jP4e1f/8kfj) to accomplish the projection as follows: we first trained a neighbors classifier on the *H* matrix obtained from LIGER for RNA alone, *H_RNA_*, (with the number of neighbors set to 3). Then, for each cell in scATAC-seq, we applied the classifier to the corresponding row *H_ATAC_*. The classifier subsequently returned the 3 nearest cells in RNA for each cell in ATAC-seq. We then compute the projections by taking the mean Decipher v component value for each cell, as well as the mean projected value onto the trajectory (Decipher time). The annotation for a given cell was simply taken to be the mode cell type of the neighbors (**Fig. S14c**).

Because this projection step may be noisy, we also implemented a filtering step wherein we computed the maximum pairwise Euclidean distance using *Scipy* [*[108]*](https://paperpile.com/c/jP4e1f/TRD6) of the Decipher space coordinates in each set of 3 nearest neighbors. The cells with distances greater than a selected threshold were removed. This step was particularly important for those Decipher embeddings that take on a curved or horseshoe shape, where a cell from ATAC-seq may be assigned to cells originating from two opposing regions.

Finally, we again implemented a nearest-neighbor smoothing to obtain a better signal for visualizations. We first built a nearest neighbors graph (k=50) on the ATAC peaks matrix. Then, the accessibility values for a given cell are obtained by taking the mean gene score for each gene across all neighbors (**Fig. S14d**)

**Comparison of scRNA-seq and scATAC-seq in the Decipher space**

For key transcription factors and marker genes, we sought to compare the patterns in their expression and their accessibility. For the expression plots, we combined the original unfiltered scRNA data for each patient's AML sample as well as the healthy bone marrow sample. We normalized these samples together by median library size and applied a natural log transformation. It was necessary to return to the unfiltered data for this task since the highly variable gene filter was applied before Decipher removed some genes of interest. We then subsetted the cells to match those that were run through Decipher previously and plotted the expression of the selected genes (from the unfiltered set) in the previously obtained Decipher space. *Scanpy* plotting was utilized, with the colorbar range set to the .02-.09 percentiles of the data. For the scATAC-seq data, we created an AnnData object from the smoothed gene scores as described above and plotted using the projected Decipher coordinates (**Fig. S15**).

**Motif Analysis in scATAC-seq**

To obtain the set of TFs for motif analysis, we examined the time of maximum expression in each of the top 50 disrupted TFs (according to the combined disruption metric). If the time of maximum expression was before the estimated time of NPM1 mutation (e.g., at pseudotime 50 for AML1), we designated the TF to be "early peaking." We then investigated whether the early peaking TFs could be putative regulators of other disrupted TFs, explaining the propagation of dysregulated mechanisms leading to the altered transcriptional landscape (**Fig. 6b,c**). Potential targets were thus defined as the set of remaining TFs (peaking after NPM1 mutation). Motif searching was performed using motifmatchR [[109]](https://paperpile.com/c/jP4e1f/3ne9) with the JASPAR2020 [[110]](https://paperpile.com/c/jP4e1f/ECkB) database of motifs and the Hg38 genome.

For each patient, we identified clusters obtained from the ArchR Iterative LSI analysis that corresponded to either blast 0 (AML1,3) or blast 1 (AML2). The ArchR reproducible peak set for that cluster, containing pseudo-bulked peak data, was used as the input to motifmatchR. To transform the result into a matrix of motifs by targets, we grouped motif scores by the target's nearest gene and calculated the mean score for all scores belonging to the same gene.

To evaluate the significance of motif enrichment, we defined a background (null) set of TFs as follows: for each patient scRNA-seq data, we filtered out genes expressed in fewer than 1 percent of cells. This threshold was selected based on the proportion of PROM1-expressing cells in the patient samples, e.g., 125 out of 13834 cells in AML3, since we expected PROM1 to be a good example of a gene expressed in the rare immature cells of interest.

We then limited the null set to TFs that were not among the target set. P-values were computed by bootstrapping: we sampled the null set 10,000 times for a subset of TFs that were the same size as the target set. We then computed the mean motif score for all TFs in the null set to create the null distribution. The p-value was obtained by counting the number of samples in the null set whose score was greater than the mean motif score of our target set of TFs. We reported the p-values for the TFs that were significantly enriched in either all three patients (in which case the maximum p-value was reported) or significantly enriched in two out of three patients (in which case the maximum among the two significant p-values was reported).

In all three patients, we found significant motif enrichment in HOX TFs, including HOXB4 (motif JASPAR ID MA1499.1, p < 0.0458) and HOXB6 (MA1500.1, p < 0.032) and FOSL1 (MA0477.1, p < 0.0057; MA0477.2 0.0086), suggesting their putative role in regulating TFs driving AML transformation (\textbf{Fig. 6d}).

In two out of three patients, we found significant motif enrichments in GATA2 (MA0036.1, p < 0.0115; MA0036.2, p < 0.0423; MA0036.3, p < 0.0309), MYC (MA0147.3, p < 0.045), NR4A2 (MA0160.1, p < 0.0272), MAFF (MA0495.1, p < 0.0365), ATF3 (MA0605.2, p < 0.0316), NFKB2 (MA0778.1, p < 0.0292), RELB (MA1117.1, p < 0.0109), KLF2 (MA1515.1, p < 0.0468), ZNF274 (MA1592.1, p < 0.009) (**Fig. S15**).
